# Interstitial Lung Disease in Antineutrophil Cytoplasmic Antibody–Associated Vasculitis: A European Multicenter Study

**DOI:** 10.1002/art.70078

**Published:** 2026-03-13

**Authors:** Aglaia Chalkia, Marusa Kotnik, Timothy J. Sadler, Spyridon Katechis, Rachel Jones, Ajay Kamath, Aladdin J. Mohammad, Sara Monti, Chetan Mukhtyar, Viral Nanda, Ioannis Petrakis, Dimitrios Petras, Ashnish Sinha, Pasupathy Sivasothy, Rona Smith, Kostas Stylianou, Dimitrios Vassilopoulos, Judith Babar, David Jayne

**Affiliations:** ^1^ Department of Medicine University of Cambridge United Kingdom; ^2^ Nephrology Department General Hospital of Athens Hippokration Athens Greece; ^3^ Department of Radiology Cambridge University Hospitals NHS Foundation Trust Cambridge United Kingdom; ^4^ Department of Radiology University of Cambridge Cambridge United Kingdom; ^5^ Rheumatology and Clinical Immunology Unit, 4th Department of Internal Medicine Attikon University Hospital and School of Medicine, National and Kapodistrian University of Athens Athens Greece; ^6^ Vasculitis and Lupus Clinic Cambridge University Hospitals NHS Foundation Trust Cambridge United Kingdom; ^7^ Department of Respiratory Medicine Norfolk and Norwich University Hospitals NHS Foundation Trust Norwich United Kingdom; ^8^ Department of Clinical Sciences Skåne University Hospital and Lund University Lund Sweden; ^9^ Department of Rheumatology Skåne University Hospital Lund Sweden; ^10^ Unit of Immunology, Allergology and Rheumatology, IRCCS Istituto Auxologico Italiano Milan Italy; ^11^ Department of Clinical Sciences and Community Health University of Milan Milan Italy; ^12^ Vasculitis Service, Rheumatology Department Norfolk and Norwich University Hospital NHS Trust Norwich United Kingdom; ^13^ Nephrology Department University General Hospital of Heraklion Crete Greece; ^14^ Clinical Immunology‐Rheumatology Unit, 2nd Department of Medicine and Laboratory, School of Medicine National and Kapodistrian University of Athens and General Hospital of Athens Hippokration Athens Greece

## Abstract

**Objective:**

Interstitial lung disease (ILD) can occur in association with antineutrophil cytoplasmic antibody (ANCA)–associated vasculitis (AAV‐ILD) or as an isolated entity with positive ANCA (ANCA‐ILD). However, data on the epidemiology and outcomes of these conditions remain limited.

**Methods:**

A European multicenter retrospective study encompassed patients with AAV‐ILD or ANCA‐ILD. Baseline and subsequent chest computed tomography studies were centrally reviewed. Primary outcomes included forced vital capacity % (FVC%) decline, respiratory failure, and mortality.

**Results:**

Of 162 patients (myeloperoxidase [MPO]–ANCA 85%), 123 (76%) had AAV‐ILD and 39 (24%) ANCA‐ILD. At baseline, usual interstitial pneumonia (UIP) was the most frequent radiologic pattern (57%), whereas half had a radiologic fibrosis grade >10%. Kidney involvement was present in 73%, most commonly Berden focal class. UIP and nonspecific interstitial pneumonia (NSIP) patterns showed greater annual FVC% decline than other patterns (UIP −1.99%, NSIP −3.76% [*P* = 0.35], others +0.36%). An adjusted mixed‐effects model indicated that rituximab was associated with mean FVC% improvement at 12 months (+6.02%; *P* = 0.07). Radiologic progression occurred in ~50%, mainly in younger patients with a higher fibrosis severity grade. Respiratory failure (19%) was associated with fibrosis severity (grade 4 hazard ratio [HR] 4.7, *P* = 0.029) and baseline FVC% (HR 0.95, *P* = 0.002). Over a median 4.2‐year follow‐up, 48% died. Age (HR 1.08, *P* = 0.04) and baseline FVC% (HR 0.97, *P* = 0.05) were independent predictors of mortality.

**Conclusion:**

At baseline, higher fibrosis severity, UIP, and lower FVC% were associated with worse outcomes. Immunosuppressives, such as rituximab, may help preserve lung function. The need for early identification and individualized treatment in ILD associated with AAV or ANCA is underscored.

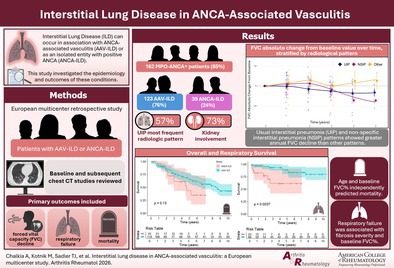

## Graphical abstract

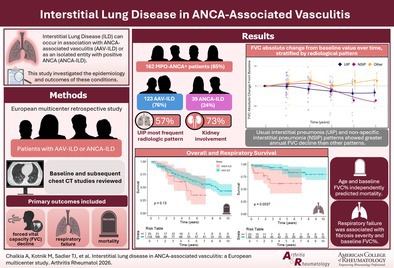

## INTRODUCTION

Antineutrophil cytoplasmic antibody (ANCA)–associated vasculitides (AAV)—including microscopic polyangiitis (MPA), granulomatosis with polyangiitis (GPA), and eosinophilic GPA (EGPA)—are small‐vessel necrotizing diseases marked by myeloperoxidase (MPO)/proteinase 3 (PR3)–ANCA autoantibodies and neutrophil‐predominant inflammation. AAV frequently involves the lungs and kidneys, with pulmonary manifestations ranging from alveolar hemorrhage and granulomatous nodules to interstitial lung disease (ILD).^1^ ILD is particularly common in MPO‐ANCA–positive patients and may occur independently of vasculitis.^2^ Prevalence varies due to heterogeneity in study designs and patient cohorts.

The pathogenesis of ILD remains unclear, though hypotheses include neutrophil‐ and macrophage‐driven inflammation in association with MPO‐ANCA and release of neutrophil extracellular traps.^3^, ^4^ Genetic factors, such as the MUC5B variant (rs35705950T), suggest overlap with other fibrotic lung diseases.^5^, ^6^ ILD in AAV is associated with poor outcomes, influenced by AAV subtype and radiologic pattern, yet treatment guidance remains limited.^7^, ^8^ The immunosuppressants routinely used for remission induction in AAV, cyclophosphamide (CYC) and rituximab (RTX), have been used to slow ILD progression with mixed results.^9^ Antifibrotic agents (pirfenidone, nintedanib) remain understudied in AAV, with most clinical trials excluding this population.^10^ This underscores the need for targeted research and evidence‐based treatment strategies.

## METHODS

### Patients

This multicenter retrospective study included patients from six centers across four European countries (Cambridge University Hospitals, Norfolk and Norwich University Hospitals, General Hospital of Athens Hippokration, University Hospital of Heraklion, Skåne University Hospital, Lund University, Fondazione IRCCS Policlinico San Matteo) with ILD associated with AAV (AAV‐ILD) or ILD with MPO/PR3–ANCA positivity without vasculitis (ANCA‐ILD). All patients had at least six months of follow‐up and a baseline chest computed tomography (CT) scan. Patients with other vasculitis diagnoses, other autoimmune diseases, or alternative ILD causes were excluded. Patient inclusion was completed up to December 2023.

### Ethics

The study complied with Good Clinical Practice and the Declaration of Helsinki. It was approved by Health Research Association and Health and Care Research Wales (IRAS ID: 33655, REC ref: 24/PR/1370) and sponsored by Cambridge University Hospitals. Local ethics approval was obtained at each country before data collection.

### Data collection

We collected demographic and clinical data from medical records, including age at diagnosis, sex, clinical phenotype, ANCA serotype, respiratory symptoms, organ involvement, immunosuppressive treatments, and estimated glomerular filtration rate (eGFR) using the Chronic Kidney Disease Epidemiology Collaboration 2021 formula. Treatment decisions followed standardized protocols or were determined through multidisciplinary team meetings across the participating centers. ILD diagnosis date was defined by the first pathologic chest CT scan (baseline), and total follow‐up was calculated from time of AAV or ANCA‐ILD diagnosis to last visit until December 2023 or death. Histopathologic features on the lung biopsy sample were included if available.

Lung function tests recorded at baseline and follow‐up included forced vital capacity (FVC%), diffusing capacity for carbon monoxide corrected (DLco_c_%), forced expiratory volume in 1 second (FEV_1_%), and total lung capacity (TLC%). FVC% decline was assessed by absolute changes. Treatment effects were evaluated by comparing FVC% from pretreatment (±3 months) to 12 months post treatment for CYC, RTX, or mycophenolate mofetil (MMF). Changes in FVC% were categorized as follows: major progression (>10% decline), moderate progression (5%–10% decline), stable (<5% change), or improvement (≥5% increase). Respiratory failure was defined as long‐term oxygen dependence of more than three months.

### Chest CT studies

The majority of the chest CT studies from diagnosis and follow‐up were centrally reviewed by specialist thoracic radiologists at Cambridge University Hospitals, who reached consensus on each study, independent of clinical data. Radiologists with a specialist interest in thoracic imaging classified the pattern of ILD according to established radiologic criteria for these diseases^11^, ^12^: (1) usual interstitial pneumonia (UIP), including definite and probable; (2) nonspecific interstitial pneumonia (NSIP); (3) other patterns, including organizing pneumonia (OP), interstitial lung abnormality (ILA), and postinflammatory scarring; and (4) overlap of the previous three. The definition of ILA followed the criteria established by the Fleischner Society.^13^ Fibrosis severity was graded using a five‐point grade based on the percentage of total bilateral lung volume involved, modified from previously published semiquantitative visual scoring systems that derive overall fibrosis extent by averaging percentage‐based assessments across defined lung areas.^14^, ^15^, ^16^ The fibrosis severity was graded as follows: grade 0 (no fibrosis), grade 1 (<5%), grade 2 (5%–10%), grade 3 (10%–25%), and grade 4 (>25%). Radiologic progression was assessed by comparing the first and last CT studies for unequivocal change in the fibrosis severity grade and pattern.

### Statistical analysis

Continuous variables, presented as means ± SDs for normally distributed data and as medians (interquartile ranges [IQRs]) for nonparametric distributions, were compared using the *t*‐test or the Mann–Whitney test, where appropriate. Categorical variables, presented as n (percentage), were compared with the chi‐square test. Longitudinal associations between FVC% and baseline exposures were examined using linear mixed‐effects models with random intercepts and slopes. A separate mixed‐effects model was used to compare pre‐ and posttreatment FVC%, adjusting for age, sex, and treatment naivety. Time‐to‐event analyses for overall and respiratory survival employed Kaplan–Meier curves and Cox proportional hazards models, with right‐censoring for patients lost to follow‐up or event free at the end of observation. Model selection used likelihood ratio tests and the Akaike information criterion. Radiologic progression was assessed with logistic regression. Analyses were performed in R (v4.4.2) using the lme4 package.

## RESULTS

### Clinical characteristics total cohort

A total of 162 patients with ILD were included in the study (Supplemental Figure S1). Of these, 123 (76%) had AAV‐ILD and 39 (24%) had ANCA‐ILD. The majority patients with AAV (n = 90, 61%) were classified as having MPA, followed by GPA (n = 23, 14%), and EGPA (n = 1, 1%). The median age at diagnosis was 72 years (IQR 65–79 years), and 57% (n = 93) of the patients were male. MPO‐ANCA was present in 85% (n = 137), and PR3‐ANCA was present in 15% (n = 25). Common symptoms at ILD diagnosis were cough and dyspnea; pulmonary hemorrhage occurred in 15%, whereas 15% were asymptomatic, with ILD detected incidentally (Table 1).

**Table 1:** Clinical characteristics of the total cohort*

Characteristics	Total cohort (N = 162)	AAV‐ILD (n = 123)	ANCA‐ILD (n = 39)	*P* value
Demographics				
Sex, % (n)				
Female	43 (69)	41 (44)	46 (18)	0.711
Male	57 (93)	59 (72)	54 (21)	–
Age at AAV diagnosis, median (IQR), y	72 (65–79)	72 (63–79)	72 (66–78)	0.903
Smoking history^a^				
Ex‐/current smoker, % (n)	57 (92)	63 (70)	56 (22)	0.567
BMI, median (IQR)^b^	29 (23–32)	28 (23–31)	31 (27–34)	0.064
Serologic phenotype, % (n)				1.00
MPO‐ANCA	85 (137)	84 (104)	85 (33)	
PR3‐ANCA	15 (25)	16 (19)	15 (6)	
ANCA levels at diagnosis, mean ± SD^c^				
MPO‐ANCA (0.0–3.4 IU/mL)	89 ± 199	103 ± 227	49 ± 50	**0.029**
PR3‐ANCA (0.0–1.9 IU/mL)	101 ± 168	134 ± 185	10 ± 5	**0.014**
Clinical phenotype, % (n)				
MPA	61 (99)	61 (99)	–	–
GPA	14 (23)	14 (23)	–	–
EGPA	1 (1)	1 (1)	–	–
Organ involvement, % (n)^d^				
Kidney	73 (86)	73 (86)	–	–
ENT	21 (25)	21 (25)	–	–
Eyes	13 (15)	13 (15)	–	–
Skin	15 (18)	15 (18)	–	–
Heart	2 (2)	2 (2)	–	–
GI	2 (2)	2 (2)	–	–
PNS	18 (21)	18 (21)	–	–
Respiratory symptoms at diagnosis, % (n)^e^				
None	15 (21)	17 (19)	7 (2)	0.368
Cough	62 (85)	57 (63)	81 (22)	**0.026**
Dyspnea	54 (75)	48 (53)	81 (22)	**0.002**
Pleuritic pain	1 (2)	2 (2)	0 (0)	1.000
Asthma	2 (3)	3 (3)	0 (0)	1.000
Diffuse alveolar hemorrhage	15 (20)	18 (20)	0 (0)	**0.013**
Lung function test at baseline, median (IQR)				
FVC%^f^	88 (71–102)	91 (74–105)	85 (66–101)	0.079
TLCOc%^g^	60 (45–74)	59 (48–73)	62 (39–77)	0.852
FEV_1_%^h^	102 (95–109)	100 (94–109)	105 (101–109)	0.185
TLC%^i^	85 (68–94)	90 (75–93)	63 (56–80)	**0.006**
Radiologic pattern at baseline, % (n)^j^				**0.019**
UIP	56 (57)	51 (39)	69 (18)	
NSIP	17 (17)	14 (11)	23 (6)	
Others	27 (28)	34 (26)	8 (2)	
Fibrosis severity grade at baseline, % (n)^k^				**0.031**
0: no fibrosis	8 (8)	9 (7)	4 (1)	
1: <5% fibrosis	27 (28)	30 (24)	14 (4)	
2: 5%–10% fibrosis	35 (36)	35 (28)	31 (8)	
3: 10%–25% fibrosis	24 (24)	19 (15)	35 (9)	
4: >25% fibrosis	11 (11)	9 (7)	15 (4)	
Immunosuppressive treatment, % (n)^l^				
Induction treatment				**<0.001**
Cyclophosphamide	51 (82)	60 (70)	31 (12)	
Rituximab	16 (26)	21 (24)	5 (2)	
Rituximab + cyclophosphamide	10 (17)	14 (16)	3 (1)	
MMF	10 (16)	5 (6)	26 (10)	
GCs	86 (140)	100 (117)	59 (23)	
No treatment	9 (14)	0 (0)	36 (14)	
Maintenance treatment				
Rituximab	41 (50)	45 (50)	–	
MMF/AZA/MTX	49 (60)	55 (60)	–	
Switch treatment				**0.002**
Cyclophosphamide	3 (5)	7 (2)	38 (3)	
Rituximab	74 (28)	87 (26)	25 (2)	
Other (MMF, AZA)	3 (5)	7 (2)	38 (3)	
Antifibrotic agent	2 (4)	3 (3)	3 (1)	1.00
Follow‐up, median (IQR), y	4.2 (2–8)	4.7 (2–9)	3 (2–5)	**<0.001**
End‐stage kidney disease, % (n)	9 (14)	11 (14)	0	**0.023**
Respiratory failure, % (n)	19 (31)	17 (21)	26 (10)	0.242
Mortality, % (n)	48 (78)	50 (62)	56 (22)	0.583
Radiologic progression, % (n)^m^	53 (55)	55 (42)	50 (13)	0.821

* Bold *P* values indicate <0.05. AAV, ANCA‐associated vasculitis; ANCA, antineutrophil cytoplasmic antibody; AZA, azathioprine; BMI, body mass index; EGPA, eosinophilic granulomatosis with polyangiitis; ENT, ear, nose, throat; FEV_1_, forced expiratory volume in 1 second; FVC%, forced vital capacity %; GCs, glucocorticoids; GI, gastrointestinal; GPA, granulomatosis with polyangiitis; ILD, interstitial lung disease; IQR, interquartile range; MMF, mycophenolate mofetil; MPA, microscopic polyangiitis; MPO, myeloperoxidase; MTX, methotrexate; NSIP, nonspecific interstitial pneumonia pattern; PNS, peripheral nervous system; PR3, proteinase 3; TLC, total lung capacity; TLCOc, carbon monoxide transfer factor corrected for hemoglobin; UIP, usual interstitial lung disease.

^a^ Twelve missing values.

^b^ Fifty‐two missing values.

^c^ Seven missing values.

^d^ Five missing values.

^e^ Twenty‐four missing values.

^f^ Forty‐six missing values.

^g^ Sixty‐four missing values.

^h^ Ninety‐six missing values.

^i^ One hundred twenty‐five missing values.

^j^ One hundred two available values.

^k^ One hundred two available values.

^l^ Thirteen missing values.

^m^ One hundred two available values.

### Radiologic and Histopathologic findings

**Figure 1:**
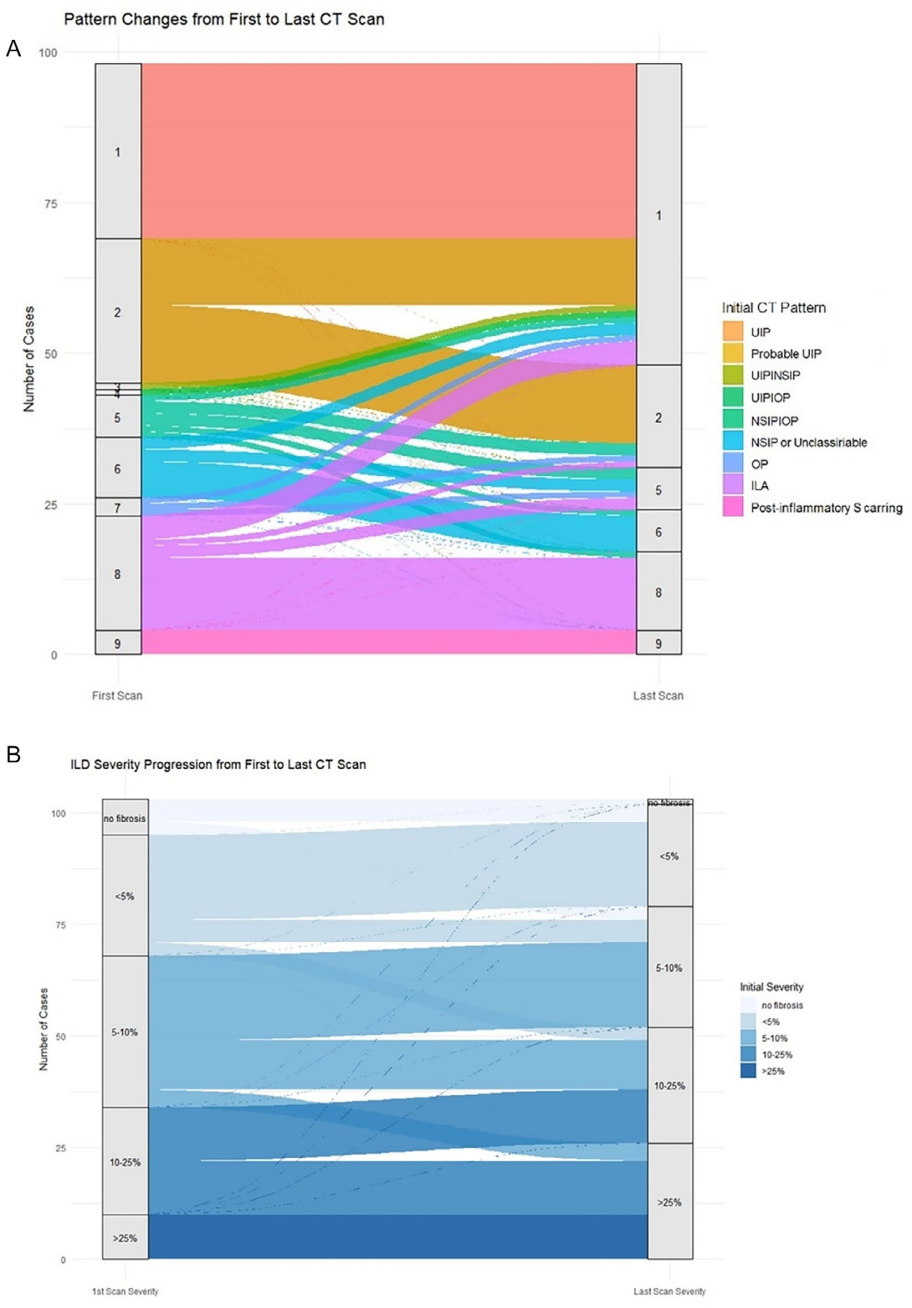
(A) Sankey diagram illustrating CT pattern changes between the first and last chest CT study. The y‐axis represents the number of cases, whereas the x‐axis tracks the transition from the first to the last CT study. The width of each bar is proportional to the number of patients with each pattern transition. Bar 1, UIP; bar 2, probable UIP; bar 3 UIP/NSIP; bar 4, UIP/OP; bar 5, NSIP/OP; bar 6, NSIP or unclassifiable; bar 7, OP; bar 8, ILA; bar 9, postinflammatory scarring. 32% of the study population had a change in radiologic pattern. (B) Sankey diagram illustrating fibrosis percent changes from the first to the last chest CT study. The y‐axis represents the number of cases, whereas the x‐axis tracks the transition from the first to the last CT study. The width of each bar is proportional to the number of patients with each severity grade transition. 38% of the patients progressed to a higher fibrosis severity grade. CT, computed tomography; ILA, interstitial lung abnormality; NSIP, nonspecific interstitial pneumonia; OP, organized pneumonia; UIP, usual interstitial pneumonia.

On the baseline chest CT scan, UIP was the most common pattern, seen in 57 patients (56%), followed by NSIP in 17 (17%) and other patterns (OP, ILA, postinflammatory scarring) reported in 28 (27%) (Table 1, Supplemental Table S1). Fibrosis severity was assessed using a five‐point grade: 8% of patients were categorized as having grade 0; 27%, grade 1; 35%, grade 2; 24%, grade 3; and 11%, grade 4. During a median follow‐up of 2.8 years (IQR 1–6 years), 53% (55 of 103) showed radiologic progression, marked by increased UIP prevalence (66%) and worsening fibrosis. The fibrosis severity increased in 38% of patients, and 32% had a change in CT pattern (Figure 1, Supplemental Table S1). Notably, all patients with definite UIP at baseline maintained this pattern over time. Among those with probable UIP, 54% progressed to definite UIP, whereas 29% of NSIP cases and 27% of other baseline patterns also evolved into a UIP pattern. Lung biopsies (n = 8) showed predominantly lymphocytic inflammation, occasional macrophages, neutrophils, giant cells, and necrosis in one case. Fibrosis was the main histopathologic feature (Supplemental Table S2).

### Kidney involvement in AAV‐ILD

Among 123 patients with AAV‐ILD, kidney involvement was the most common extrapulmonary manifestation, affecting 86 (73%) patients (Supplemental Table S3). The median age was 75 (IQR 72–79) years. At baseline, the most common radiologic pattern was UIP (49%), and the fibrosis severity was as follows: 4%, grade 0; 33%, grade 1; 31%, grade 2; 22%, grade 3; and 8%, grade 4. The median eGFR was 29 mL/min per 1.73 m^2^ (IQR 13–49 mL/min per 1.73 m^2^), with 25 patients (29%) having an eGFR <15 mL/min per 1.73 m^2^. Albuminuria, measured as a urine albumin‐to‐creatinine ratio of >300 mg/g, was seen in 63% of patients, and 87% had hematuria. Kidney biopsy was performed in 64 patients; the most common Berden class was focal (47%), followed by mixed (31%), crescentic (17%), and sclerotic (5%). The median percentage of normal glomeruli in each biopsy was 50% (IQR 23–72), cellular crescents 9% (IQR 0–29), and global sclerosis 12% (IQR 2–28). The majority of the patients showed absent or mild tubulointerstitial fibrosis in the kidney biopsy (72%). Over a median follow‐up period of 5 years (IQR 2–9 years), 13 progressed to end‐stage kidney disease, whereas half had radiologic progression of ILD.

### ANCA‐ILD vs AAV‐ILD

Demographics were similar between the groups (Table 1). Patients with ANCA‐ILD had lower MPO and PR3 levels (*P* = 0.029 and *P* = 0.014, respectively), despite similar serotype distribution. At baseline, respiratory symptoms, particularly cough and dyspnea, were more common in ANCA‐ILD (*P* = 0.026, *P* = 0.002). At baseline, FVC% was comparable, but TLC was lower in ANCA‐ILD (*P* = 0.006). ANCA‐ILD more frequently showed a UIP pattern (69% vs 51%; *P* = 0.019) and higher fibrosis grades (*P* = 0.031). Immunosuppressive treatment, particularly glucocorticoids and induction regimens, was used more commonly in AAV‐ILD (*P* < 0.001). Mortality, respiratory failure, and radiologic progression were similar across both groups. During the follow‐up period, no patient with ANCA‐ILD progressed to AAV‐ILD.

### MPO‐ANCA vs PR3‐ANCA

Of the total cohort (N = 162), 137 had MPO‐ANCA and 25 had PR3‐ANCA. Demographics were similar between the groups (Supplemental Table S4). MPO‐ANCA was associated with MPA, whereas PR3‐ANCA was linked to GPA, with more ear, nose, and throat and skin involvement (*P* = 0.023, *P* = 0.008, *P* = 0.004). Regarding the respiratory symptoms at diagnosis, patients with PR3‐ANCA were more symptomatic compared to patients with MPO‐ANCA (100% vs 82%; *P* = 0.043). FVC% was similar, though patients with PR3‐ANCA had lower FEV_1_% and higher TLC% (*P* = 0.012, *P* = 0.029). UIP was more common in patients with MPO‐ANCA (61% vs 40%; *P* = 0.006), with a trend toward greater fibrosis severity. Treatment regimens and outcomes were comparable.

### Pulmonary function decline

**Figure 2:**
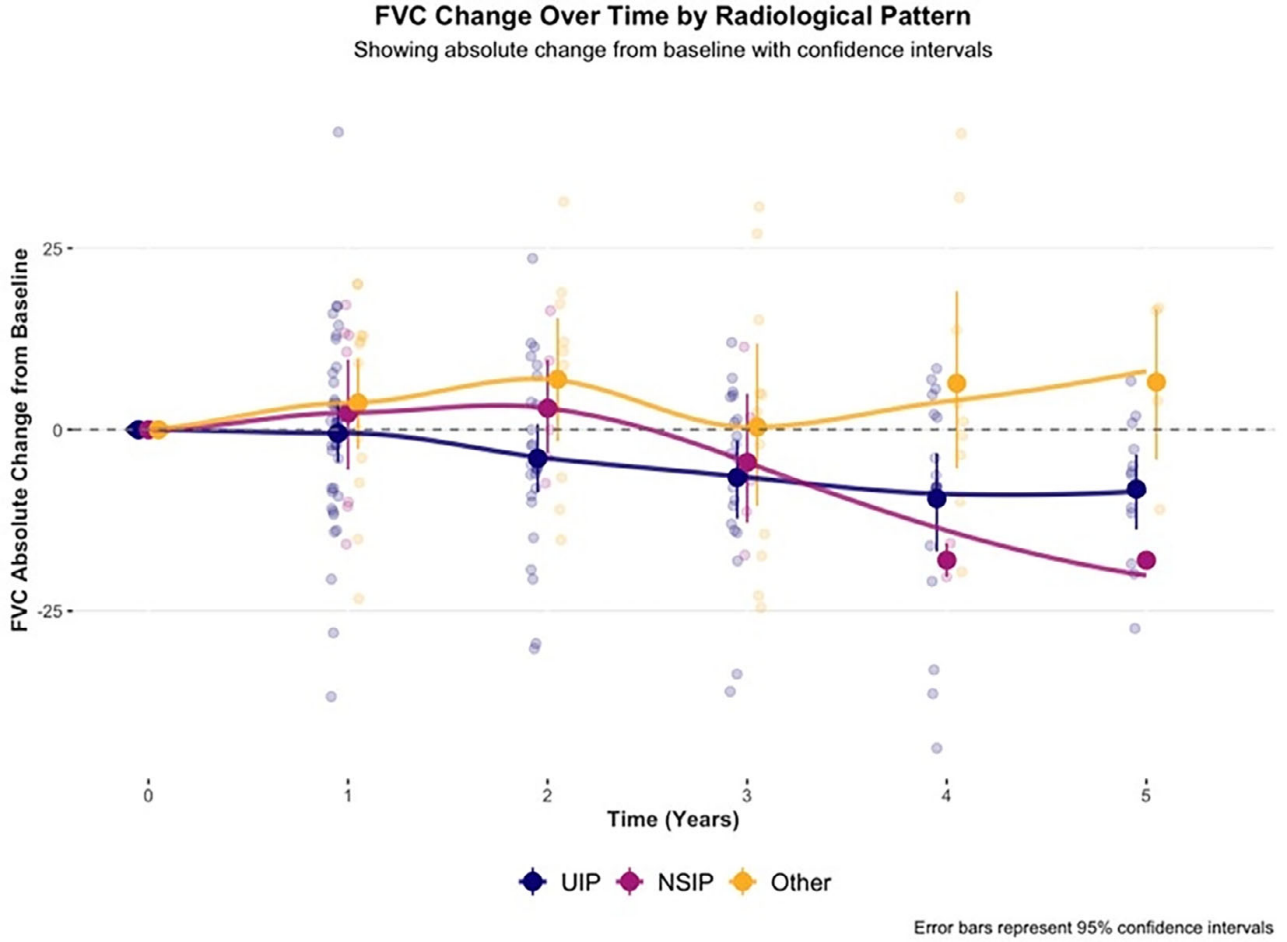
FVC% absolute change from baseline value over time, stratified by radiologic pattern. Longitudinal analysis of absolute FVC% change over a five‐year period. Points represent marginal mean values, as estimated by a mixed‐effects model, at each time point, and vertical bars represent 95% confidence intervals. Individual patient data are shown as background dots. The UIP and NSIP groups exhibited a declining trend, whereas other patterns demonstrated relatively stable or improved FVC% values over time. FVC%, forced vital capacity %; NSIP, nonspecific interstitial pneumonia; UIP, usual interstitial pneumonia.

Sequential FVC% data were available for 116 patients (72%) over a median follow‐up of 4.2 years (IQR 2–8 years). Baseline FVC% did not differ between the radiologic patterns (Table 1, Figure 2). Changes in FVC% from baseline over time for the three radiologic categories (UIP, NSIP, other patterns) are shown in Figure 2.

Annual FVC% changes by radiologic pattern were as follows: UIP, −1.99% (95% confidence interval [CI] −2.9 to −1.0, *P* < 0.001); NSIP, −3.76% (95% CI −6.1 to −1.4, *P* = 0.002); other, +0.36% (95% CI −1.0 to 1.7, *P* = 0.62). Both UIP and NSIP showed greater FVC% decline compared to other patterns (UIP −2.35%, *P* = 0.02; NSIP −4.12%, *P* = 0.01), with no difference between UIP and NSIP (*P* = 0.35).

Within group comparisons assessed whether radiologic pattern was associated with FVC% decline over time. In the UIP pattern, we observed declines from baseline at year 4 (adjusted difference −8.34%, 95% CI −14 to −3, *P* = 0.01) and year 5 (adjusted difference −9.18%, 95% CI −15 to −3, *P* = 0.02). In contrast, no within‐group FVC% changes were observed in the NSIP or other patterns groups.

Between‐group comparisons evaluated whether the FVC% decline differed between radiologic subtypes. Compared to patients with other patterns, those with UIP demonstrated greater FVC% decline as early as year 1 (adjusted difference −16.6%, 95% CI −28 to −6, *P* = 0.009), which persisted through year 5 (adjusted difference −22.8%, 95% CI −37 to −9, *P* = 0.005). Similarly, NSIP was associated with a greater decline than other patterns by year 5 (adjusted difference −32.1%, 95% CI −56 to −8, *P* = 0.02).

### Radiologic progression

Radiologic progression was observed in 53% of the cohort (55 of 103) (Supplemental Table S5). Patients with radiologic progression were younger (*P* = 0.014). Radiologic progressors tended to present more often with a UIP pattern (64% vs 48%; *P* = 0.069) and were less likely to have the lowest fibrosis severity grade at baseline (29% vs 40%; *P* = 0.004). Although radiologic progressors and nonprogressors had similar overall FVC% decline by year 5 (−8.88% vs −8.97%; *P* = 0.931), progressors presented a more consistent and sustained trajectory of functional deterioration (Supplemental Figure S2).

### Immunosuppressive treatment

In the total cohort, 96% of the patients received immunosuppressive treatment. In the ANCA‐ILD group, 59% received glucocorticoids, 31% received CYC, 5% received RTX, 3% received combination therapy (CYC + RTX), 26% received other agents (MMF, azathioprine, methotrexate, leflunomide), and 38% received no immunosuppression. All patients with AAV‐ILD received glucocorticoids; 60% received CYC, 21% received RTX, 14% received combination therapy (CYC + RTX), and 5% received MMF as induction therapy, with RTX (46%) and MMF/azathioprine (54%) for maintenance (Table 1).

**Figure 3:**
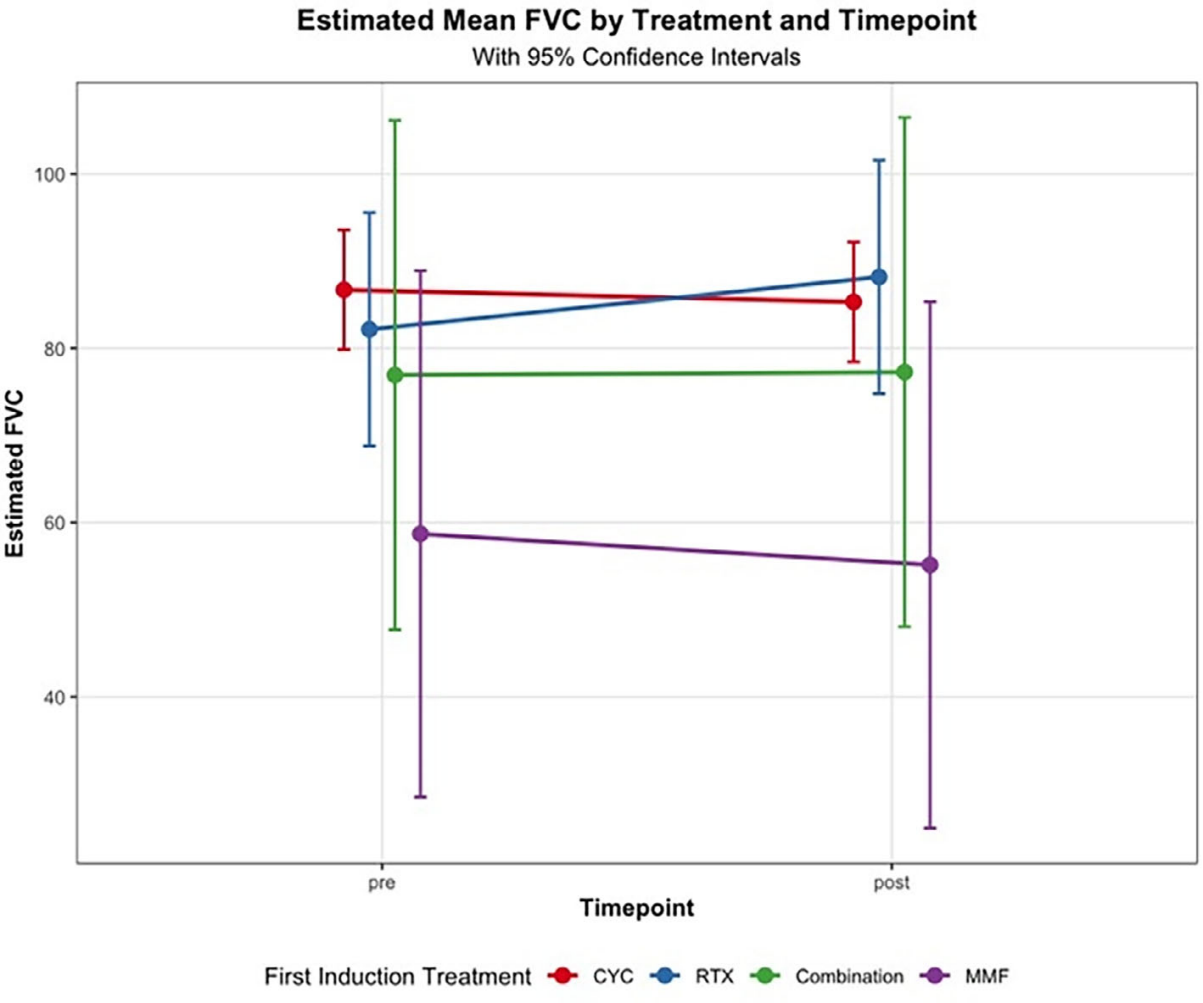
Estimated mean FVC% changes before and 12 months after treatment initiation. Dots represent the marginal mean values, as estimated by a mixed‐effects model adjusted for age, sex, and treatment naivety, with vertical lines representing 95% confidence intervals, for patients who received different therapies. combination, CYC + RTX; CYC, cyclophosphamide; FVC%, forced vital capacity %; MMF, mycophenolate mofetil; RTX, rituximab.

Using an adjusted mixed‐effects model, mean FVC% change at 12 months post induction was as follows: CYC, −1.39% (95% CI −4.18 to 1.41, *P* = 0.33); RTX, +6.02% (95% CI −0.44 to 12.48, *P* = 0.07); combination therapy (CYC + RTX), +0.35% (95% CI −8.79 to 9.49, *P* = 0.94); and MMF, −3.57% (95% CI −16.49 to 9.35, *P* = 0.59) (Figure 3). Baseline lung function and CT patterns were similar across treatment groups (Supplemental Table S6). In the total cohort, RTX showed the greatest annual FVC% gain: +3.07% per year (95% CI 0.67–5.47) (Supplemental Table S7). Notably, in the subgroup analysis of patients with a UIP pattern (n = 51), those who received RTX (n = 17) showed a greater increase in FVC% (+13.8; *P* = 0.013) (Supplemental Figure S3). Among patients with NSIP (n = 12) or other patterns (n = 17), RTX appeared to help stabilize lung function, though without significant differences (Supplemental Figure S4). In additional subgroup analysis, RTX also showed a consistent trend toward improved annual FVC% in both ANCA‐ILD (n = 20) and AAV‐ILD (n = 71) (Supplemental Figure S4).

### Antifibrotic treatment

Four patients (3%; three men, one woman) received antifibrotics (one received pirfenidone, three received nintedanib). All were MPO‐ANCA positive: three with AAV‐ILD and one with ANCA‐ILD. AAV‐ILD cases showed renal involvement (eGFR 41 mL/min/1.73 m^2^), diffuse alveolar hemorrhage, cutaneous, or peripheral nervous system manifestations. All received CYC plus glucocorticoids. Baseline CT showed UIP in 75% of patients (fibrosis severity grade 4 in 75%, grade 3 in 25%); one had NSIP/OP. The median FVC% was 72 (range 44–72). At one year, half declined 5% to 10% in FVC% and half were stable. Over 2.3 years (range 2–5 years), two died and two developed respiratory failure.

### Clinical outcomes and poor risk factors

#### FVC% and radiologic progression

After one year of treatment, FVC% progression was observed as followed: major progression (FVC% decline >10%) in 21% of patients (18 of 85), moderate progression (FVC% decline 5%–10%) in 8% of patients (7 of 85), stable or minimal improvement (<5% change in FVC%) in 38% of patients (32 of 85), and improvement (FVC% increase >5%) in 33% of patients (28 of 85). Logistic regression identified kidney involvement as the only predictor of major FVC% decline, with an odds ratio (OR) of 3.08 (95% CI 1.03–10.1) (Supplemental Table S8).

Fifty‐three percent of the cohort (55 of 103) showed radiologic progression. Younger age (OR 0.94, 95% CI 0.89–0.98, *P* = 0.015) and greater baseline fibrosis severity (grade 4: OR 0.04, 95% CI 0.00–0.35, *P* = 0.012) were associated with a higher likelihood of progression (Supplemental Table S9).

#### Respiratory failure

**Figure 4:**
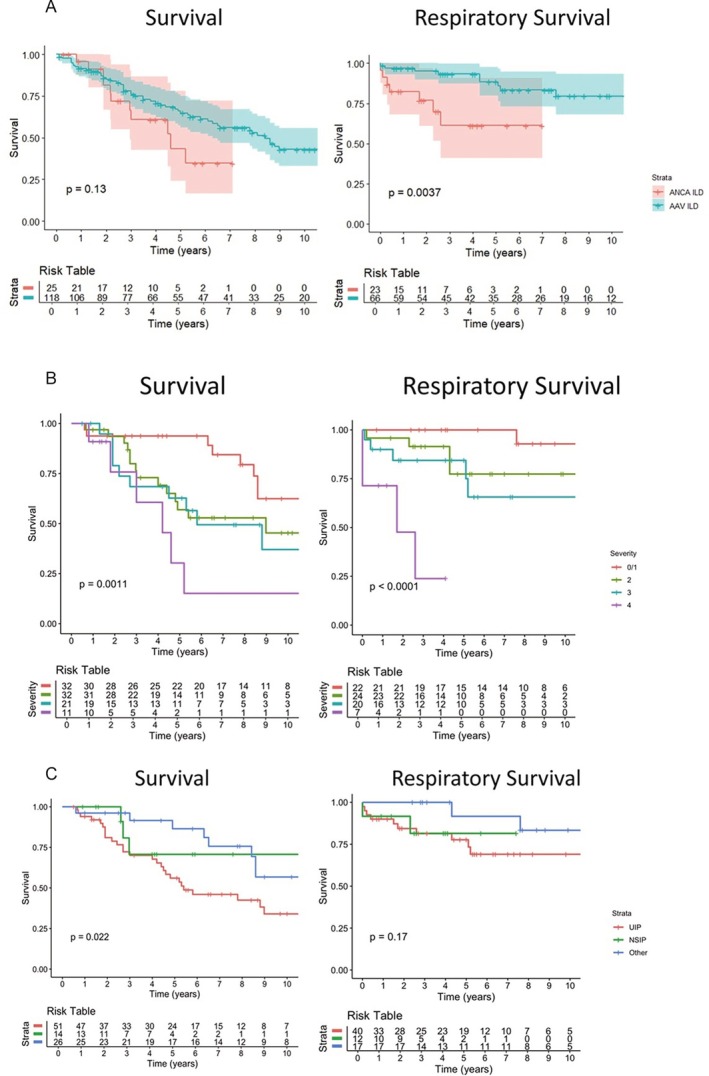
Overall and respiratory survival analyses. (A) Kaplan–Meier survival curves comparing the overall (left) and respiratory (right) survival of patients with ANCA‐ILD (red) and AAV‐ILD (blue). Shade areas represent estimated 95% confidence intervals. *P* values represent the respective log‐rank test. Overall survival did not differ between the two groups; patients with ANCA‐ILD demonstrated worse respiratory survival (*P* < 0.01). (B) Kaplan–Meier survival curves comparing overall (left) and respiratory (right) survival across different fibrosis severity groups. *P* values represent the respective log‐rank test. Fibrosis severity was associated with lower overall (*P* < 0.01) and respiratory (*P* < 0.0001) survival, with a grade of 4 exhibiting the worse outcomes. (C) Kaplan–Meier survival curves comparing overall (left) and respiratory (right) survival of patients with UIP (red), NSIP (green), or other (blue) radiologic ILD patterns. *P* values represent the respective log‐rank test. Overall survival was lower in the UIP group (*P* < 0.05). Respiratory survival did not differ (*P* = 0.17). AAV, ANCA‐associated vasculitis; ANCA, antineutrophil cytoplasmic antibody; ILD, interstitial lung disease; NSIP, nonspecific interstitial pneumonia; UIP, usual interstitial pneumonia.

Over a median follow‐up of 4.2 years (IQR 2–8 years), 31 patients (19%) developed respiratory failure, defined as long‐term oxygen dependence. Median respiratory failure–free survival time was 18.2 years (95% CI 11.6–20), with 5‐ and 10‐year survival rates of 82.1% and 74.3%, respectively. Respiratory failure occurred earlier in the ANCA‐ILD group compared to the AAV‐ILD group (log‐rank *P* = 0.003; Figure 4A). Although radiologic patterns showed no differences, higher baseline fibrosis grades were associated with worse respiratory outcomes (*P* < 0.001; Figure 4B, Supplemental Table S10). In univariate analysis, predictors included fibrosis severity, ILD subtype, induction or antifibrotic treatment, and baseline FVC% and TLC (Supplemental Table S11). Multivariable analysis identified two independent risk factors: baseline FVC% (hazard ratio [HR] 0.95, 95% CI 0.92–0.98, *P* = 0.002) and grade 4 fibrosis (HR 4.7, 95% CI 1.17–19.1, *P* = 0.029; Table 2).

**Table 2:** Multivariable Cox regression analysis of factors associated with overall survival and respiratory survival*

Variables	Survival			Respiratory survival		
	HR	95% CI	*P* value	HR	95% CI	*P* value
Age at AAV diagnosis	1.08	1.03 to 1.14	**0.004**	1.02	0.96 to 1.09	0.43
Sex						
Male (ref)	–	–	–	–	–	–
Female	0.64	0.28 to 1.46	0.29	1.49	0.45 to 5.00	0.51
ANCA type						
MPO (ref)	–	–	–	–	–	–
PR3	1.78	0.49 to 6.54	0.38	0.00	0.00 to inf	>0.99
ILD group						
ANCA‐ILD (ref)	–	–	–	–	–	–
AAV‐ILD	0.43	0.15 to 1.26	0.12	0.53	0.15 to 1.82	0.31
Radiologic pattern						
UIP (ref)	–	–	–	–	–	–
NSIP	0.54	0.12 to 2.47	0.43	–	–	–
Others	0.47	0.07 to 3.30	0.45	–	–	–
Fibrosis severity grade						
1 (ref)	–	–	–	–	–	–
2	1.91	0.32 to 11.4	0.48	1.69	0.45 to 6.34	0.44
3	2.13	0.29 to 15.5	0.46	2.43	0.78 to 7.59	0.13
4	3.48	0.42 to 29	0.25	4.72	1.17 to 19.1	**0.029**
FVC% predicted baseline	0.97	0.95 to 0.99	**0.005**	0.95	0.92 to 0.98	**0.002**
Kidney involvement (no ref)	1.08	0.44 to 2.67	0.87	1.04	0.30 to 3.64	0.95

* Bold *P* values indicate <0.05. AAV, ANCA‐associated vasculitis; ANCA, antineutrophil cytoplasmic antibody; CI, confidence interval; FVC%, forced vital capacity %; HR, hazard radio; ILD, interstitial lung disease; MPO, myeloperoxidase; NSIP, nonspecific interstitial pneumonia pattern; PR3, proteinase 3; UIP, usual interstitial pneumonia.

#### Mortality

Over a median follow‐up of 4.2 years (IQR 2–8 years), 48% (n = 78) of patients died. Median survival was 8.1 years (95% CI 5.8–11.9), with 5‐ and 10‐year survival rates of 61.9% and 40.6% respectively. Survival did not differ between the AAV‐ILD and ANCA‐ILD groups (log‐rank *P* = 0.13; Figure 4A). The UIP pattern was associated with worse survival than NSIP or other patterns (*P* = 0.022; Figure 4C), and higher baseline fibrosis grades predicted poorer outcomes (*P* = 0.001; Figure 4B). A fibrosis grade 4 was associated with a nearly six‐fold increase in mortality risk (HR 5.8, 95% CI 2.1–15.7, Supplemental Table S10). Lung disease progression—due to infection or ILD exacerbation—was the leading cause of death. Other infections were rare; two deaths were attributed to lung cancer (Supplemental Table S12). Univariate Cox analysis identified several mortality predictors: age, respiratory failure, radiologic pattern, induction treatment, ANCA levels, and baseline FVC%, DLco_c_%, and TLC (Supplemental Table S11). On multivariate analysis, age at diagnosis (HR 1.08, 95% CI 1.03–1.14, *P* = 0.004) and baseline FVC% (HR 0.97, 95% CI 0.95–0.99, *P* = 0.005) were independent predictors of mortality (Table 2).

## DISCUSSION

In this European multicenter cohort of patients with AAV‐ILD and ANCA‐ILD, we identified meaningful clinical, radiologic, and prognostic features that help define this underrecognized disease spectrum. The majority of patients were MPO‐ANCA positive and presented with a UIP pattern. Rates of radiologic progression and functional decline were substantial, especially in patients with UIP and in those with severe fibrosis. Baseline pulmonary function, particularly FVC%, and the severity of radiologic fibrosis emerged as independent predictors of both mortality, which was high, with lung disease progression as a leading cause of death, and respiratory failure. Although immunosuppressive treatments were widely used, RTX showed a trend toward improved lung function. Importantly, kidney involvement was common but did not impact overall survival, highlighting respiratory decline as the primary driver of mortality in this population. Given the limited data from previous retrospective studies and the absence of standardized treatment approaches, our study offers real‐world insights that may inform clinical practice and future research directions.

In our cohort, the majority (85%) of patients were MPO‐ANCA positive, consistent with prior studies—particularly from Asia—highlighting the strong association between MPO‐ANCA, MPA, and pulmonary fibrosis.^17^, ^18^ Although the underlying mechanisms are unclear, MPO‐ANCA might contribute to alveolar injury and fibrosis via neutrophil or macrophage activation and oxidative stress. Furthermore, the distinct clinical phenotypes have been reported between ANCA‐positive and ANCA‐negative vasculitis, in which the latter typically presents with more renal‐limited disease and less pulmonary involvement, suggesting a potential pathogenic role of ANCA antibodies in alveolar injury.^19^ In our cohort, the UIP pattern was more common with MPO‐ANCA than PR3‐ANCA, with a trend toward greater fibrosis severity. Although FVC% was comparable between groups, PR3‐ANCA showed lower FEV_1_% and higher TLC%, consistent with a previous report.^20^ Lung histopathologic studies in AAV‐ILD are limited^20^, ^21^; however, our findings support fibrosis as the dominant pathologic process. Histopathologic studies in both AAV‐ILD and ANCA‐ILD are warranted to better elucidate disease mechanisms and identify potential therapeutic targets.

Recurrent or subclinical lung injury may initiate a sustained proinflammatory response, contributing to both pulmonary fibrosis and vasculitis.^22^, ^23^ We identified UIP as the predominant radiologic pattern (57%), consistent with previous AAV‐ILD reports.^7^, ^18^ Notably, over half of the patients had a radiologic fibrosis grade exceeding 10% at baseline—this being, to our knowledge, the first study to quantify radiologic fibrosis burden in AAV‐ILD using a standardized scoring system. Both ANCA‐ILD and AAV‐ILD showed similar baseline pulmonary function, though fibrosis was more severe in ANCA‐ILD, likely reflecting earlier ILD onset, consistent with previous report.^24^ Despite these differences, long‐term outcomes were comparable, supporting the rationale for a unified therapeutic strategy. Although up to 25% of patients with ANCA‐ILD have been reported to progress to MPA,^24^, ^25^ no such progression was observed in our cohort. Conversely, 19% of patients with AAV‐ILD had a history of ILD, in which the absence of earlier ANCA testing may represent a diagnostic pitfall. Nevertheless, the clinical utility and cost‐effectiveness of routine ANCA testing in patients with suspected idiopathic pulmonary fibrosis remain uncertain and warrant further investigation.

Kidney involvement in AAV‐ILD is well recognized, though data on its histopathologic features and long‐term outcomes remain limited.^17^ In our cohort, kidney involvement was common (73%) predominantly in patients with MPO‐ANCA positivity and a concurrent UIP pattern. Kidney biopsies typically revealed pauci‐immune glomerulonephritis, most often within the focal and mixed classes of the Berden classification, corresponding to a low risk of progression according to ANCA Kidney Risk Score criteria.^26^ Although MPO‐ANCA is strongly linked to glomerulonephritis and renal fibrosis,^27^ we did not observe an association between ILD and kidney fibrosis. One plausible explanation is the earlier clinical recognition and treatment of renal disease due to more overt symptoms, whereas ILD can progress subclinically until advanced fibrosis occurs. Despite a generally favorable renal prognosis in our cohort, over half of the patients experienced radiologic progression of ILD, and overall mortality remained high. These findings suggest that respiratory complications are a stronger predictor of mortality than renal involvement and progress independently of kidney disease and that the treatment responses of ILD and kidney vasculitis are quite different.

At baseline, the median FVC% was modestly reduced, with no differences across radiologic subtypes, consistent with a prior report.^18^ Despite similar initial lung function, FVC% trajectories diverged over time; patients with a UIP pattern experienced the most pronounced and rapid decline, followed by those with NSIP, underscoring the prognostic value of radiologic subtype. A major FVC% decline (>10%) at one year was observed in 21% of patients—an established marker of poor prognosis in idiopathic pulmonary fibrosis^28^—slightly lower than previously reported in AAV‐ILD.^29^ Approximately half of the cohort remained stable radiologically and functionally, whereas the remainder—predominantly younger patients and greater baseline fibrosis severity—progressed. Although a higher progression rate (87.5%) has been reported, that finding was based on a small sample.^9^ In our study, all patients with baseline UIP maintained this pattern, whereas approximately 30% of those initially diagnosed with NSIP or other patterns demonstrated progression to a UIP pattern, highlighting the dynamic and evolving nature of radiologic manifestations in ANCA‐ILD. These findings highlight the urgent need for reliable biomarkers and clinical predictors of progression, as both radiologic pattern and fibrosis extent appear to offer practical prognostic value in guiding management and risk stratification in ANCA‐ILD.

Most patients in our cohort received immunosuppressive therapy, primarily glucocorticoids combined with CYC or RTX. Although effective for inducing remission in vasculitis, the impact of these agents on ILD progression remains unclear.^30^, ^31^ RTX was associated with a trend toward improved FVC% at one year compared to CYC (alone or combined) or MMF, consistent with limited prior findings in AAV.^9^ Although the sample size limited the power of subgroup analyses, the apparent benefit of RTX was consistent across both ANCA‐ILD and AAV‐ILD subgroups and appeared most pronounced in patients with a UIP pattern. Similarly, in other connective tissue disease–associated ILDs (CTD‐ILDs), such as rheumatoid arthritis and scleroderma‐related ILD, RTX has been shown to stabilize or even improve lung function across both UIP and non‐UIP patterns, suggesting that the disease course and underlying pathogenesis in CTD‐ILDs differ from those in idiopathic ILD.^32^ However, the apparent benefit observed with RTX in our cohort may be influenced by treatment selection bias, with CYC preferentially used in more severe systemic disease. Whether the benefit of RTX persists with continued therapy, or whether specific antifibrotic treatment is required, remains unknown. Similarly, the effect of the complement C5a receptor inhibitor, avacopan, on ILD has not been studied; none of the patients in our cohort received avacopan, as it was not available during the study period. Mortality and respiratory failure remained high, particularly in patients with extensive baseline fibrosis, underscoring the limited impact of current treatments.^17^ Antifibrotics (nintedanib or pirfenidone) were used in only 3% of cases, reflecting their exclusion from major trials and the absence of clear guidance in AAV‐ILD. These findings highlight the urgent need for prospective studies evaluating antifibrotics, alone or with immunosuppression, in this population.

Survival in our cohort was poor, with a 5‐year survival rate of 62% and median survival of 8.1 years, highlighting the severe prognosis associated with ILD. Our findings align with previously published cohort studies focusing specifically on AAV‐ILD.^33^, ^34^ Respiratory progression emerged as the leading cause of death, emphasizing the need for early identification and aggressive management of lung disease. Baseline FVC%, UIP pattern, and fibrosis severity were independent predictors of mortality and respiratory failure, reinforcing the prognostic importance of early and accurate radiologic and functional assessment. Notably, no survival differences were observed between patients with AAV‐ILD and those with ANCA‐ILD, suggesting that fibrotic burden and radiologic pattern may carry greater prognostic weight than the presence of systemic vasculitis.

This study has several limitations. The retrospective, observational design introduces risks of selection bias, incomplete data, and variable follow‐up. Heterogeneity in imaging protocols and pulmonary function testing across centers may have influenced radiologic and functional assessments, and treatment was nonrandomized, reflecting real‐world clinical practice. Limited use of antifibrotic agents restricts conclusions on their efficacy, and the inclusion of patients from specialized ILD referral centers may limit generalizability. Nevertheless, this represents one of the largest European cohorts of patients with AAV‐ILD and ANCA‐ILD, with centralized CT re‐evaluation enhancing diagnostic consistency and standardized fibrosis scoring. Longitudinal assessment of radiologic progression, alongside epidemiologic and functional data, provides novel insights into disease natural history and prognostic factors, highlighting the need for prospective trials in this underrecognized population.

ILD is an increasingly recognized manifestation of AAV, frequently presenting with a UIP pattern and substantial fibrotic burden. These features were associated with radiologic progression, functional decline, and increased mortality. Notably, baseline pulmonary function and the extent of radiologic fibrosis emerged as independent predictors of adverse outcomes, whereas renal involvement—despite being prevalent—did not impact overall survival. Immunosuppressive therapy demonstrated limited efficacy in halting respiratory progression, particularly RTX, and the use of antifibrotic agents was minimal, underscoring existing uncertainties regarding their role. These findings highlight the urgent need for early diagnosis, standardized radiologic evaluation, and a multidisciplinary strategy to optimize care and improve prognosis in this underrecognized, high‐risk patient population. Future advances will rely on the integration of clinical, molecular, and imaging data to elucidate disease mechanisms and guide precision therapies in AAV‐ILD. Prospective multicenter studies exploring B cell–targeted and complement‐targeted treatments, in combination with antifibrotic, are essential to translate these insights into improved patient care.

## AUTHOR CONTRIBUTIONS

All authors contributed to at least one of the following manuscript preparation roles: conceptualization AND/OR methodology, software, investigation, formal analysis, data curation, visualization, and validation AND drafting or reviewing/editing the final draft. As corresponding author, Dr Jayne confirms that all authors have provided the final approval of the version to be published and takes responsibility for the affirmations regarding article submission (eg, not under consideration by another journal), the integrity of the data presented, and the statements regarding compliance with institutional review board/Declaration of Helsinki requirements.

## Supporting information


**Disclosure form**.

**Appendix S1:** Supplementary Information
