# Supplementary material for: Interstitial Lung Disease in Antineutrophil Cytoplasmic Antibody–Associated Vasculitis: A European Multicenter Study
Source: Arthritis Rheumatol. 2026 Mar 13;78(8):1718–30. doi: 10.1002/art.70078 (PMC13430081; doi:10.1002/art.70078)
Supplement: Supplementary file 2 — Appendix S1: Supplementary Information [file ART-78-1718-s001.docx]

**Interstitial Lung Disease in ANCA-Associated Vasculitis: A European Multicentre Study**

Supplementary File

**Supplemental Figure S1.** Flow chart of the study

**Supplemental Table S1.** Radiological patterns and Severity in CT studies

**Supplemental Table S2.** Histopathological characteristics of lung biopsy

**Supplemental Table S3.** Kidney involvement in AAV-ILD

**Supplemental Table S4.** Clinical characteristics between MPO and PR3-ANCA positive

**Supplemental Table S5**. Clinical characteristics between Radiological progressors and non-radiological progressors

**Supplemental Figure S2.** Force Vital Capacity (FVC) change over time among radiological progressors and non-radiological progressors

**Supplemental Table S6**. Clinical characteristics between treatment groups

**Supplemental Table S7.** Mean annual change in Force Vital Capacity (FVC) by induction treatment

**Supplemental Figure S3.** Estimated mean Force Vital Capacity (FVC) changes before and 12 months after treatment initiation in patients with UIP pattern

Supplemental Figure S4. Estimated mean Force Vital Capacity (FVC) changes before and 12 months after treatment initiation in patients with NSIP pattern (a), other pattern (b), ANCA-ILD (c), AAV-ILD (d)

**Supplemental Table S8.** Logistic Regression for FVC progression (FVC decline>10%) after the first year

**Supplemental Table S9**. Logistic Regression for radiological progression

**Supplemental Table S10.** Univariate Cox regression of fibrosis severity grade with overall and respiratory survival

**Supplemental Table S11.** Univariate Cox regression of overall and respiratory survival

**Supplemental Table S12.** Causes of death

**Supplemental Figure S1.** Flow chart of the study


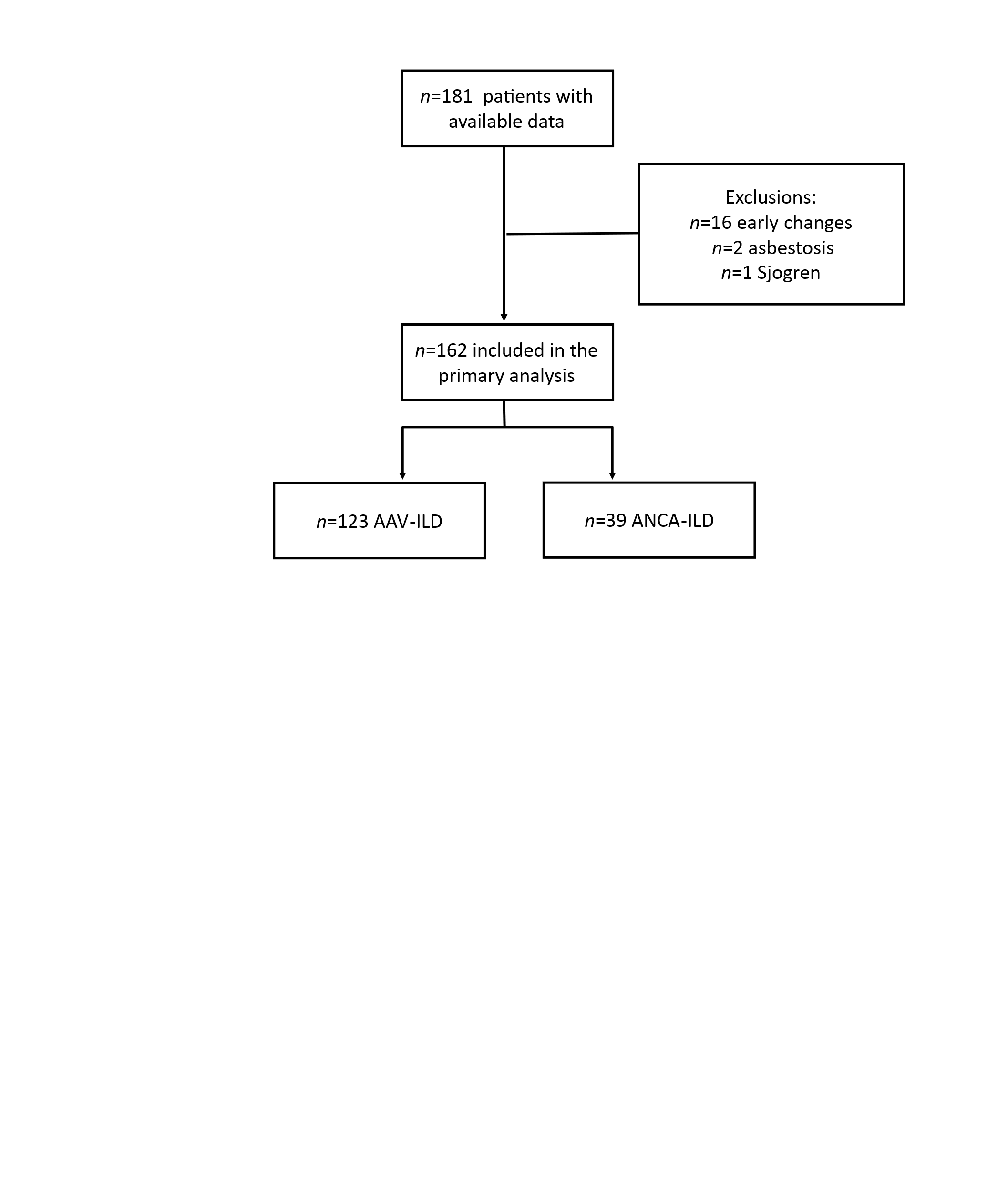


**Supplemental Table S1.** Radiological patterns and Severity in CT studies

|  | **ILD cohort** | |
| --- | --- | --- |
|  | **Baseline CT study**  ***n*=102** | **Last CT study**  ***n*=103** |
| **Radiological pattern**  **Usual Interstitial Pneumonia (UIP), % (n)**  Definite UIP, % (n)  Probable UIP, % (n)  UIP/NSIP, % (n)  UIP/OP, % (n)  **Non-Specific Interstitial Pneumonia (NSIP), % (n)**  NSIP/OP, % (n)  NSIP or unclassifiable, % (n)  **Other, % (n)**  Organizing pneumonia (OP), % (n)  ILA (Interstitial Lung Abnormalities), % (n)  Post-inflammatory scarring % (n) | **57 (58)**  31 (32)  24 (24)  1 (1)  1 (1)  **17 (17)**  7 (7)  10 (10)  **24 (27)**  3 (3)  20 (20)  4 (4) | **66 (68)**  **49** (50)  18 (18)  0 (0)  0 (0)  **16 (16)**  8 (8)  8 (8)  **19 (20)**  0 (0)  15 (15)  4 (4) |
| **Fibrosis Severity Grade**  0 no fibrosis, % (n)  1 <5% fibrosis, % (n)  2 5-10% fibrosis, % (n)  3 10-25% fibrosis, % (n)  4 >25% fibrosis, % (n) | 8 (8)  27 (28)  35 (36)  24 (24)  11 (11) | 1 (1)  23 (23)  26 (27)  24 (25)  24 (25) |
| **Radiological Progression** | 53 (55)  2.8 (0.9-6) | |
| **Internal time 1^st^-last CT study years, median (IQR)** |  |  |

ILD; interstitial lung disease, n; number, IQR; interquartile range, UIP, usual interstitial lung disease, NSIP; Non-Significant Interstitial Pneumonia pattern,

**Supplemental Table S2.** Histopathological characteristics of lung biopsy

| **Lung Biopsy-**  **Histopathological characteristics** | **Radiological Pattern** | **Clinical**  **Characteristics** |
| --- | --- | --- |
| Non-specific interstitial pneumonia (NSIP) | Non-specific interstitial pneumonia (NSIP) | MPO-ANCA  AAV-ILD |
| Mild non-specific focal abnormalities | Non-specific interstitial pneumonia (NSIP) | MPO-ANCA  AAV-ILD |
| Fibrotic organized pneumonia. Central peribronchial vascular fibrosis. | Non-specific interstitial pneumonia (NSIP) | MPO-ANCA  ANCA-ILD |
| Fibrinoid tissue necrosis associated with granulomatous inflammation with giant cells, neutrophils, eosinophils, plasma cells and lymphocytes. There is also fibrinoid necrosis in a small artery. | Other (post-inflammatory scarring) | PR3-ANCA  AAV-ILD |
| Mild nonspecific chronic inflammation. No evidence of vasculitis. | Other (post-inflammatory scarring) | MPO-ANCA  AAV-ILD |
| Mild infiltrate of lymphocytes and plasma cells in the lamina propria and rarely neutrophils. No vascular necrosis is found. | Probable UIP | PR3-ANCA  AAV-ILD |
| Non-necrotising granuloma is seen and with increased numbers of alveolar macrophages.  A mild mixed inflammatory infiltrate is seen in the bronchial mucosa and occasional small vessels show infiltration by neutrophils.  There is no necrosis. | Other (Interstitial lung abnormalities) | MPO-ANCA  ANCA-ILD |
| Usual interstitial pneumonia (UIP). Patchy fibrosis adjacent to areas with normal alveolar structure. Fibroblastic foci are seen at the margins of areas of established fibrosis. There are small collections of foamy histiocytes in alveolar spaces but no granulomas. | Probable UIP | MPO-ANCA  AAV-ILD |

ILD; interstitial lung disease, AAV; ANCA-associated Vasculitis, MPO; Myeloperoxidase, PR3; proteinase 3;

**Supplemental Table S3.** Kidney involvement in AAV-ILD

| **Characteristics** | **AAV-ILD with kidney involvement**  ***n*=86** |
| --- | --- |
| **Demographics**  **Gender**  Female % (n)  Male % (n)  **Age at AAV diagnosis** (years), median (IQR) | 37 (32)  63 (54)  75 (72-79) |
| **Serologic phenotype**  MPO % (n)  PR3 % (n) | 91 (78)  9 (8) |
| **ANCA levels at diagnosis ^a^**  MPO-ANCA (0.0-3.4 IU/ml), median (IQR)  PR3-ANCA (0.0-1.9 IU/ml), median (IQR) | 76 (25-134)  177 (125-231) |
| **Clinical phenotype**  MPA % (n)  GPA % (n) | 84 (72)  16 (14) |
| **Respiratory symptoms at diagnosis ^b^**  None % (n)  Cough % (n)  Dyspnoea % (n)  Pleuritic pain % (n)  Asthma % (n)  Diffuse Alveolar haemorrhage % (n) | 22 (18)  57 (46)  40 (31)  2 (2)  1 (1)  21 (17) |
| **Lung Function Test at Baseline**  FVC % median (IQR) ^c^  TLCOc % median (IQR) ^d^  FEV1 % median (IQR) ^f^  TLC % median (IQR) ^e^ | 91 (77-102)  56 (45-73)  100 (94-112)  89 (73-94) |
| **Radiological pattern at Baseline ^f^**  UIP % (n)  NSIP % (n)  Others % (n) | 49 (24)  14 (7)  37 (18) |
| **Fibrosis severity grade at Baseline ^g^**  0 no fibrosis, % (n)  1 <5% fibrosis, % (n)  2 5-10% fibrosis, % (n)  3 10-25% fibrosis, % (n)  4 >25% fibrosis, % (n) | 4 (3)  33 (17)  31 (16)  22 (11)  8 (4) |
| **Kidney function at diagnosis**  eGFR ml/min/1.73m2 median (IQR)  ACR mg/mmol >300mg/g, % (n) ^h^  Haematuria % (n) ^i^ | 29 (13-49)  63 (37)  87 (60) |
| **Berden Classification ^j^**  Focal class % (n)  Mixed class % (n)  Crescentic class % (n)  Sclerotic class % (n) | 47 (30)  31 (20)  17 (11)  5 (3) |
| **AKRiS ^k^**  Low risk % (n)  Moderate % (n)  High % (n)  Very High % (n) | 82 (27)  6 (2)  12 (4)  0 (0) |
| **Glomeruli**  Glomeruli %, median (IQR)  Normal Glomeruli (%), median (IQR)  Global Sclerosis (%), median (IQR)  Cellular Crescents (%), median (IQR)  Fibrinoid Necrosis (%), median (IQR)  Fibrous Crescents (%), median (IQR) | 20 (14-18)  50 (23-72)  12 (2-28)  9 (0-29)  5 (0-14)  0 (0-1) |
| **Interstitial/tubular area ^l^**  Fibrosis/ atrophy   - Absent % (n) - Mild % (n) - Moderate % (n) - Severe % (n)   Tubulitis % (n)  Acute tubular injury % (n) | 39 (24)  34 (21)  20 (12)  7 (4)  13 (8)  54 (34) |
| **Vessels**  Arteritis % (n)  Arteriosclerosis % (n) | 16 (10)  42 (27) |
| **Immunosuppressive treatment** ^m^  Induction Treatment  Cyclophosphamide % (n)  Rituximab % (n)  Rituximab + Cyclophosphamide % (n)  MMF % (n)  Maintenance treatment  Rituximab % (n)  MMF/AZA/MTX % (n)  Antifibrotic agent % (n) | 62 (50)  21 (18)  17 (14)  5 (4)  48 (39)  57 (46)  1 (1) |
| Follow-up (years), median (IQR)  End-stage kidney disease % (n)  Respiratory failure % (n)  Mortality % (n)  Radiological progression % (n) ^n^ | 5 (2-9)  15 (13)  15 (13)  49 (42)  54 (26) |

ILD; interstitial lung disease, n; number, IQR; interquartile range, AAV; ANCA-associated Vasculitis, MPO; Myeloperoxidase, PR3; proteinase 3; MPA; Microscopic polyangiitis, GPA; Granulomatosis with polyangiitis, FVC; Forced Vital Capacity, TLCOc; carbon monoxide transfer factor corrected for hemoglobin, FEV1; forced expiratory volume in 1 second, TLC; total lung capacity, UIP, usual interstitial lung disease, NSIP; Non-Significant Interstitial Pneumonia pattern, MMF; mycophenolate mofetil, AZA; azathioprine, MTX; methotrexate, AKRiS; 2024 ANCA kidney risk score

^a^ 2 missing values

^b^ 5 missing values

^c^ 36 missing values

^d^ 41 missing values

^f^ 60 missing values

^e^ 72 missing values

^f^ 37 missing values

^g^ 35 missing values

^h^  26 missing values

^i^  16 missing values

^j^ 21 missing values

^k^ 52 missing values

^l^ 24 missing values

^m^  5 missing values

^n^ 38 missing values

**Supplemental Table S4.** Clinical characteristics of the total cohort between MPO and PR3-ANCA positive

| **Characteristics** | **Total Cohort**  ***n*=162** | **MPO-ANCA**  ***n*=137** | **PR3-ANCA**  ***n*=25** | **P value** |
| --- | --- | --- | --- | --- |
| **Demographics**  **Gender**  Female % (n)  Male % (n)  **Age at AAV diagnosis** (years), median (IQR)  **Smoking history ^a^**  Ex/current smoker % (n)  **BMI** kg/m^2^ median (IQR)^b^ | 43 (69)  57 (93)  72 (65-79)  57 (92)  29 (23-32) | 43 (59)  57 (78)  73 (65-89)  61 (77)  28 (24-31) | 40 (10)  60 (15)  69 (62-74)  63 (15)  30 (25-33) | 0.829  **0.041**  1.00  1.00 |
| **ANCA levels at diagnosis^c^**  mean ±SD |  | 89± 199 | 101±168 | 0.674 |
| **Clinical phenotype**  MPA % (n)  GPA % (n)  EGPA % (n)  No AAV % (n) | 61 (99)  14 (23)  1 (1)  24 (39) | 71 (97)  4 (6)  1 (1)  24 (33) | 8 (2)  68 (17)  0 (0)  24 (6) | **0.023** |
| **Organ involvement ^d^**  Kidney % (n)  ENT % (n)  Eyes % (n)  Skin % (n)  Heart % (n)  GI % (n)  PNS % (n) | 73 (86)  21 (25)  13 (15)  15 (18)  2 (2)  2 (2)  18 (21) | 63 (78)  14 (17)  9 (11)  9 (11)  2 (2)  1 (1)  15 (19) | 40 (8)  40 (8)  20 (4)  35 (7)  0 (0)  5 (1)  10 (2) | 0.083  **0.008**  0.227  **0.004**  1.00  0.259  0.738 |
| **Respiratory symptoms at diagnosis ^e^**  None % (n)  Cough % (n)  Dyspnoea % (n)  Pleuritic pain % (n)  Asthma % (n)  Diffuse Alveolar haemorrhage % (n) | 15 (21)  62 (85)  54 (75)  1 (2)  2 (3)  15 (20) | 18 (21)  63 (74)  51 (60)  2 (2)  1 (1)  14 (16) | 0 (0)  55 (11)  75 (15)  0 (0)  10 (2)  20 (4) | **0.043**  0.620  0.054  1.00  0.05  0.492 |
| **Lung Function Test at Baseline**  FVC % median (IQR) ^f^  TLCOc % median (IQR) ^g^  FEV1 % median (IQR) ^h^  TLC % median (IQR) ^i^ | 88 (71-102)  60 (45-74)  102 (95-109)  85 (68-94) | 87 (72-101)  60 (45-74)  104 (98-111)  81 (65-92) | 101 (70-117)  59 (52-78)  92 (90-101)  93 (90-107) | 0.144  0.714  **0.012**  **0.029** |
| **Radiological pattern at Baseline ^j^**  UIP % (n)  NSIP % (n)  Others % (n) | 56 (57)  17 (17)  27 (28) | 61 (53)  17 (15)  22 (19) | 40 (6)  0 (0)  60 (9) | **0.006** |
| **Fibrosis severity grade at Baseline ^k^**  0 no fibrosis, % (n)  1 <5% fibrosis, % (n)  2 5-10% fibrosis, % (n)  3 10-25% fibrosis, % (n)  4 >25% fibrosis, % (n) | 8 (8)  27 (28)  35 (36)  24 (24)  11 (11) | 7 (6)  22 (20)  34 (31)  26 (24)  11 (10) | 13 (2)  50 (8)  31 (5)  0 (0)  6 (1) | 0.055 |
| **Immunosuppressive treatment** ^l^  Induction Treatment  Cyclophosphamide % (n)  Rituximab % (n)  Rituximab + Cyclophosphamide % (n)  MMF % (n)  GCs % (n)  No treatment % (n)  Maintenance treatment  Rituximab % (n)  MMF/AZA/MTX % (n)  Swich treatment  Cyclophosphamide % (n)  Rituximab % (n)  Other (MMF, AZA) % (n)  Antifibrotic agent % (n) | 51 (82)  16 (26)  10 (17)  10 (16)  86 (140)  9 (14)  41 (50)  49 (60)  3 (5)  74 (28)  3 (5)  2 (4) | 51 (67)  19 (25)  11 (15)  9 (12)  90 (119)  9 (12)  45 (42)  55 (52)  16 (5)  72 (23)  13 (4)  3 (4) | 63 (15)  4 (1)  8 (2)  17 (4)  88 (21)  8 (2)  50 (8)  50 (8)  0 (0)  80 (5)  17 (1)  0 (0) | 0.344  0.788  0.579  1.00 |
| Follow-up (years), median (IQR)  End-stage kidney disease % (n)  Respiratory failure % (n)  Mortality % (n)  Radiological progression % (n) ^m^ | 4.2 (2-8)  9 (14)  19 (31)  48 (78)  53 (55) | 4 (2-8)  10 (13)  21 (28)  52 (71)  21 (28) | 4 (3-10)  4 (1)  12 (3)  52 (13)  12 (3) | 0.177  0.698  0.415  1.00  0.415 |

^a^ 12 missing values

^b^ 52 missing values

^c^  7 missing values

^d^  5 missing values

^e^  24 missing values

^f^ 46 missing values

^g^ 64 missing values

^h^ 96 missing values

^i^  125 missing values

^j^  102 available values

^k^ 102 available values

^l^  13 missing values

^m^ 102 available values

ILD; interstitial lung disease, n; number, IQR; interquartile range, AAV; ANCA-associated Vasculitis, BMI; Body mass index, MPO; Myeloperoxidase, PR3; proteinase 3; MPA; Microscopic polyangiitis, GPA; Granulomatosis with polyangiitis, EGPA; Eosinophilic granulomatosis with polyangiitis, ENT; Ear, Nose, Throat, GI; Gastrointestinal, PNS; Peripheral Nervous System, FVC; Forced Vital Capacity, TLCOc; carbon monoxide transfer factor corrected for hemoglobin, FEV1; forced expiratory volume in 1 second, TLC; total lung capacity, UIP, usual interstitial lung disease, NSIP; Non-Significant Interstitial Pneumonia pattern, MMF; mycophenolate mofetil, GCs; Glucocorticoids, AZA; azathioprine, MTX; methotrexate

**Supplemental Figure S2.** Force Vital Capacity (FVC) change over time, stratified by radiological progression


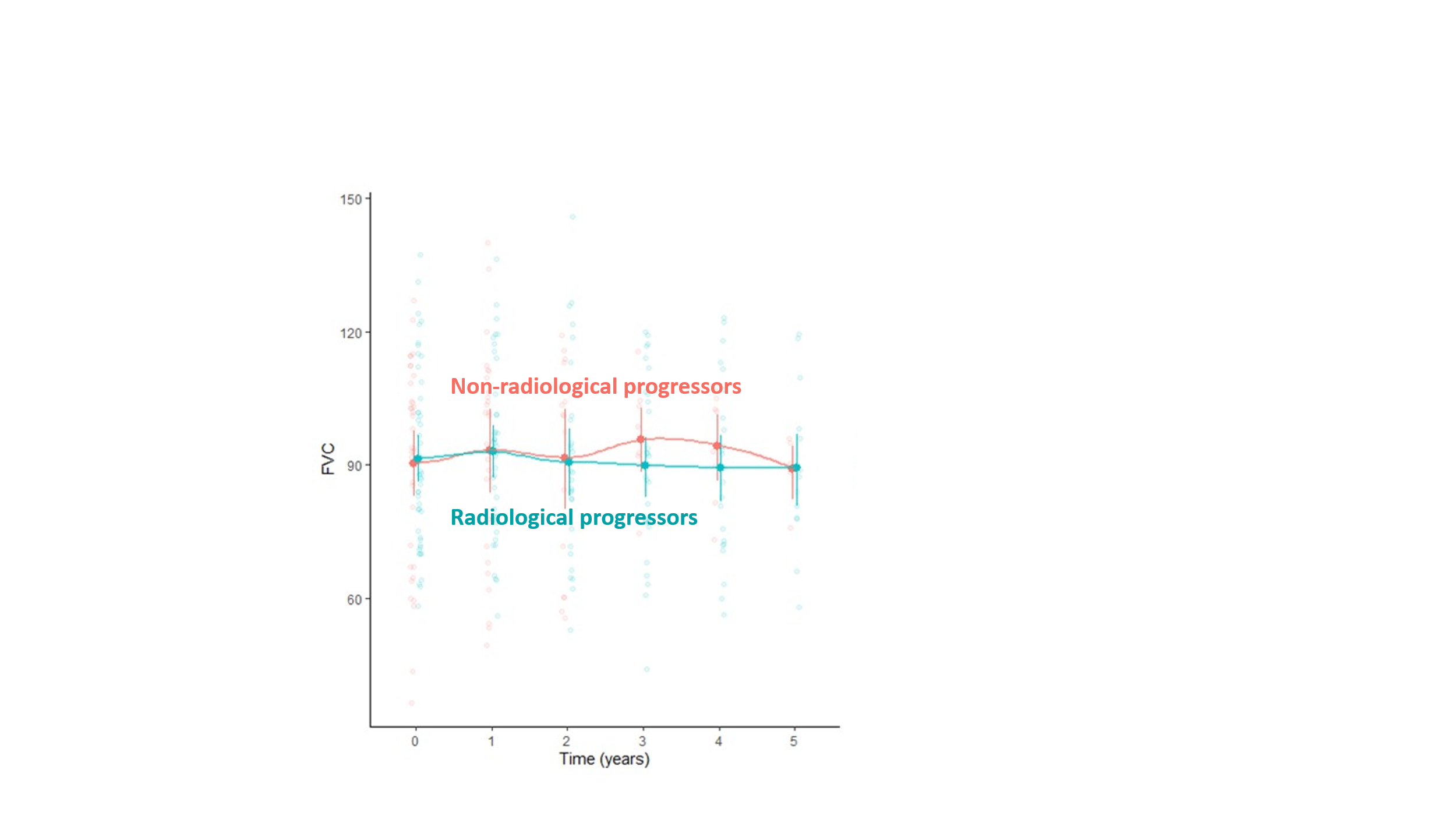


Longitudinal analysis of FVC change over a 5-year period. Points represent marginal mean values, as estimated by a mixed effects model, at each time-point and vertical bars represent 95% confidence intervals. Individual patient data are shown as background dots. FVC values did not differ significantly between the two groups.

**Supplemental Table S5**. Clinical characteristics between Radiological progressors and non-radiological progressors

| **Characteristics** | **Total Cohort**  ***n*=162** | **Radiological progressors**  ***n*=55** | **Non-radiological progressors**  ***n*=48** | **P value** |
| --- | --- | --- | --- | --- |
| **Demographics**  **Gender**  Female % (n)  Male % (n)  **Age at AAV diagnosis**  (years), median (IQR)  **Smoking history ^a^**  Ex/current smoker % (n)  **BMI** kg/m^2^ median (IQR)^b^ | 43 (69)  57 (93)  72 (65-79)  57 (92)  29 (23-32) | 44 (24)  56 (31)  69 (61-73)  61 (33)  29 (26-33) | 29 (14)  71 (34)  75 (71-79)  63 (29)  29 (25-31) | 0.154  **0.014**  1.000  0.788 |
| **Serologic phenotype**  MPO-ANCA % (n)  PR3-ANCA % (n) | 85 (137)  15 (25) | 87 (48)  13 (7) | 81 (39)  19 (9) | 0.427 |
| **Clinical phenotype**  MPA % (n)  GPA % (n)  EGPA % (n)  No AAV | 61 (99)  14 (23)  1 (1) | 64 (35)  13 (7)  0 (0)  24 (13) | 58 (28)  13 (6)  2 (1)  27 (13) | 0.709 |
| **Organ involvement ^c^**  Kidney % (n)  ENT % (n)  Eyes % (n)  Skin % (n)  Heart % (n)  GI % (n)  PNS % (n) | 73 (86)  21 (25)  13 (15)  15 (18)  2 (2)  2 (2)  18 (21) | 26/50  6/46  8/50  8/50  0/50  1/50  9/50 | 22/46  11/50  6/50  5/46  1/46  1/46  8/46 | 0.838  0.293  0.777  0.557  0.479  1.000  1.000 |
| **Respiratory symptoms at diagnosis ^d^**  None % (n)  Cough % (n)  Dyspnoea % (n)  Pleuritic pain % (n)  Asthma % (n)  Diffuse Alveolar haemorrhage % (n) | 15 (21)  62 (85)  54 (75)  1 (2)  2 (3)  15 (20) | 2/43  30/43  27/43  0/43  0/43  7/43 | 3/41  22/41  26/41  0/41  0/41  7/41 | 0.672  0.178  1.000  >0.99  >0.99  1.000 |
| **Lung Function Test at Baseline**  FVC % median (IQR) ^e^  TLCOc % median (IQR) ^f^  FEV1 % median (IQR) ^g^  TLC % median (IQR) ^h^ | 88 (71-102)  60 (45-74)  102 (95-109)  85 (68-94) | 88 (74-103)  58 (48-74)  102 (96-109)  90 (76-98) | 94 (68-107)  66 (49-80)  100 (93-109)  85 (71-92) | 0.723  0.366  0.592  0.335 |
| **Radiological pattern at Baseline ^i^**  UIP % (n)  NSIP % (n)  Others % (n) | 56 (57)  17 (17)  27 (28) | 64 (32)  14 (7)  22 (11) | 48 (23)  21 (10)  31 (15) | 0.069 |
| **Fibrosis severity grade at Baseline ^j^**  0 no fibrosis, % (n)  1 <5% fibrosis, % (n)  2 5-10% fibrosis, % (n)  3 10-25% fibrosis, % (n)  4 >25% fibrosis, % (n) | 8 (8)  27 (28)  35 (36)  24 (24)  11 (11) | 13 (7)  16 (9)  38 (21)  29 (16)  4 (2) | 2 (1)  38 (18)  27 (13)  17 (8)  17 (8) | **0.004** |
| **Immunosuppressive treatment** ^k^  Induction Treatment  Cyclophosphamide % (n)  Rituximab % (n)  Rituximab + Cyclophosphamide % (n)  MMF % (n)  GCs % (n)  No treatment % (n)  Maintenance treatment  Rituximab % (n)  MMF/AZA/MTX % (n)  Swich treatment  Cyclophosphamide % (n)  Rituximab % (n)  Other (MMF, AZA) % (n)  Antifibrotic agent % (n) | 51 (82)  16 (26)  10 (17)  10 (16)  86 (140)  9 (14)  41 (50)  49 (60)  3 (5)  74 (28)  3 (5)  2 (4) | 63 (34)  20 (11)  4 (2)  7 (4)  91 (49)  6 (3)  35 (14)  65 (26)  14 (3)  71 (15)  14 (3)  2 (1) | 56 (25)  11 (5)  13 (6)  7 (3)  89 (40)  13 (6)  55 (16)  45 (13)  13 (1)  63 (5)  25 (2)  7 (3) | 0.199  0.140  0.792  0.327 |
| Follow-up (years), median (IQR)  End-stage kidney disease % (n)  Respiratory failure % (n)  Mortality % (n) | 4.2 (2-8)  9 (14)  19 (31)  48 (78) | 6 (4-9)  5 (3)  29 (16)  53 (29) | 3 (2-7)  4 (2)  17 (8)  44 (21) | **0.007**  1.000  0.168  0.431 |

^a^ 12 missing values

^b^ 52 missing values

^c^  5 missing values

^d^  24 missing values

^e^ 46 missing values

^f^ 64 missing values

^g^ 96 missing values

^h^  125 missing values

^i^  102 available values

^j^ 102 available values

^k^  13 missing values

ILD; interstitial lung disease, n; number, IQR; interquartile range, AAV; ANCA-associated Vasculitis, BMI; Body mass index, MPO; Myeloperoxidase, PR3; proteinase 3; MPA; Microscopic polyangiitis, GPA; Granulomatosis with polyangiitis, EGPA; Eosinophilic granulomatosis with polyangiitis, ENT; Ear, Nose, Throat, GI; Gastrointestinal, PNS; Peripheral Nervous System, FVC; Forced Vital Capacity, TLCOc; carbon monoxide transfer factor corrected for hemoglobin, FEV1; forced expiratory volume in 1 second, TLC; total lung capacity, UIP, usual interstitial lung disease, NSIP; Non-Significant Interstitial Pneumonia pattern, MMF; mycophenolate mofetil, GCs; Glucocorticoids, AZA; azathioprine, MTX; methotrexate

**Supplemental Table S6**. Clinical characteristics between treatment groups

| **Characteristics** | **CYC**  ***n*=92** | **RTX**  ***n*=30** | **CYC+RTX**  ***n*=21** | **MMF**  ***n*=17** | **p value** |
| --- | --- | --- | --- | --- | --- |
| **Demographics**  **Gender**  Female % (n)  Male % (n) | 42 (39)  58 (53) | 47 (14)  53 (16) | 38 (8)  62 (13) | 41 (7)  59 (10) | 0.840 |
| **Clinical phenotype**  MPA % (n)  GPA % (n)  EGPA % (n)  Νο ΑΑV % (n) | 63 (58)  23 (21)  0 (0)  13 (12) | 83 (25)  10 (3)  0 (0)  7 (2) | 76 (16)  19 (4)  0 (0)  5 (1) | 29 (5)  12 (2)  0 (0)  59 (10) | **<0.001** |
| **Lung Function Test at Baseline**  FVC % mean ±SD  TLCOc % mean ±SD  FEV1 % mean ±SD  TLC % mean ±SD | 91 ±23  63±19  102±12  90±14 | 82±17  51±17  100±13  70±15 | 89±18  59±11  95±14  82±15 | 74±24  60±24  105±5  66±18 | 0.066  0.300  0.755  **0.006** |
| **Radiological pattern at Baseline ^a^**  UIP % (n)  NSIP % (n)  Others % (n) | 61 (35/57)  12 (7/57)  26 (15/57) | 53 (9/17)  24 (4/17)  24 (4/17) | 38 (3/8)  25 (2/8)  38 (3/8) | 43 (3/7)  14 (1/7)  43 (3/7) | 0.877 |
| **Fibrosis severity grade at Baseline ^b^**  0 no fibrosis, % (n)  1 <5% fibrosis, % (n)  2 5-10% fibrosis, % (n)  3 10-25% fibrosis, % (n)  4 >25% fibrosis, % (n) | 10 (6/61)  21 (13/61)  30 (18/61)  28 (17/61)  11 (7/61) | 6 (1/18)  22 (4/18)  44 (8/18)  11 (2/18)  17 (3/18) | 0 (0/8)  50 (4/8)  25 (2/8)  13 (1/8)  13 (1/8) | 0 (0/7)  43 (3/7)  43 (3/7)  14 (1/7)  0 (0) | 0.783 |

^a^ 89 available values

^b^ 94 available values

n; number, SD; standard deviation, MPA; Microscopic polyangiitis, GPA; Granulomatosis with polyangiitis, EGPA; Eosinophilic granulomatosis with polyangiitis, AAV; anca-associated Vasculitis, FVC; Forced Vital Capacity, TLCOc; carbon monoxide transfer factor corrected for hemoglobin, FEV1; forced expiratory volume in 1 second, TLC; total lung capacity, UIP, usual interstitial lung disease, NSIP; Non-Significant Interstitial Pneumonia pattern, CYC; cyclophosphamide, RTX; rituximab, MMF; mycophenolate mofetil

**Supplemental Table S7.** Mean annual change in Force Vital Capacity (FVC) by induction treatment

| **Mean Yearly Change in FVC by Induction Treatment** | | | | | |
| --- | --- | --- | --- | --- | --- |
| Treatment | Slope Estimate | Standard Error | DF | Lower CL | Upper CL |
| CYC | -1.922 | 0.389 | 274.441 | -2.688 | -1.156 |
| RTX | 3.074 | 1.219 | 305.927 | 0.676 | 5.472 |
| Combination (RTX+CYC) | -4.381 | 1.143 | 284.915 | -6.632 | -2.131 |
| MMF | -0.170 | 1.259 | 300.261 | -2.647 | 2.307 |
| No treatment | -1.513 | 0.938 | 288.214 | -3.360 | 0.334 |

CYC; cyclophosphamide, RTX; rituximab, MMF; mycophenolate mofetil; DF; degrees of freedom; CI=Confidence Interval

**Supplemental Figure S3.** Estimated mean Force Vital Capacity (FVC) changes before and 12 months after treatment initiation in patients with UIP pattern

**
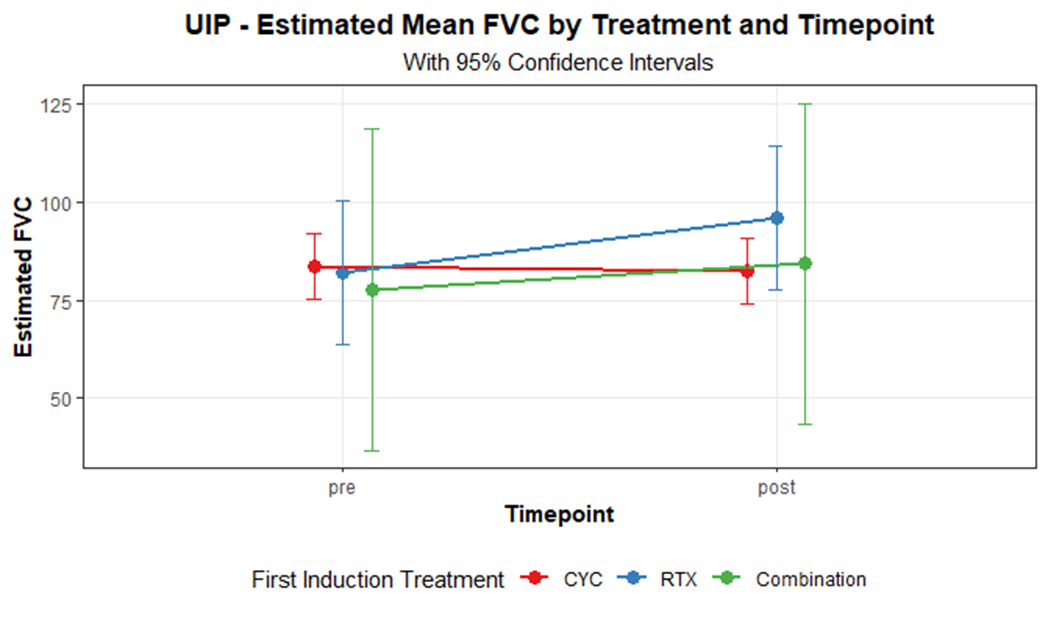
**

CYC=-1.1%, p=0.534

RTX=+13.8%, p=0.013

Combination=+6.6%, p=0.586

Dots represent the marginal mean values, as estimated by a mixed effects model adjusted for age, gender treatment naivety, with vertical lines representing 95% confidence intervals, for UIP patients that received different therapies

UIP; Usual Interstitial Pneumonia, CYC; cyclophosphamide, RTX; rituximab, combination; CYC+RTX,

Supplemental Figure S4. Estimated mean Force Vital Capacity (FVC) changes before and 12 months after treatment initiation in patients with NSIP pattern (a), other pattern (b), ANCA-ILD (c), AAV-ILD (d)


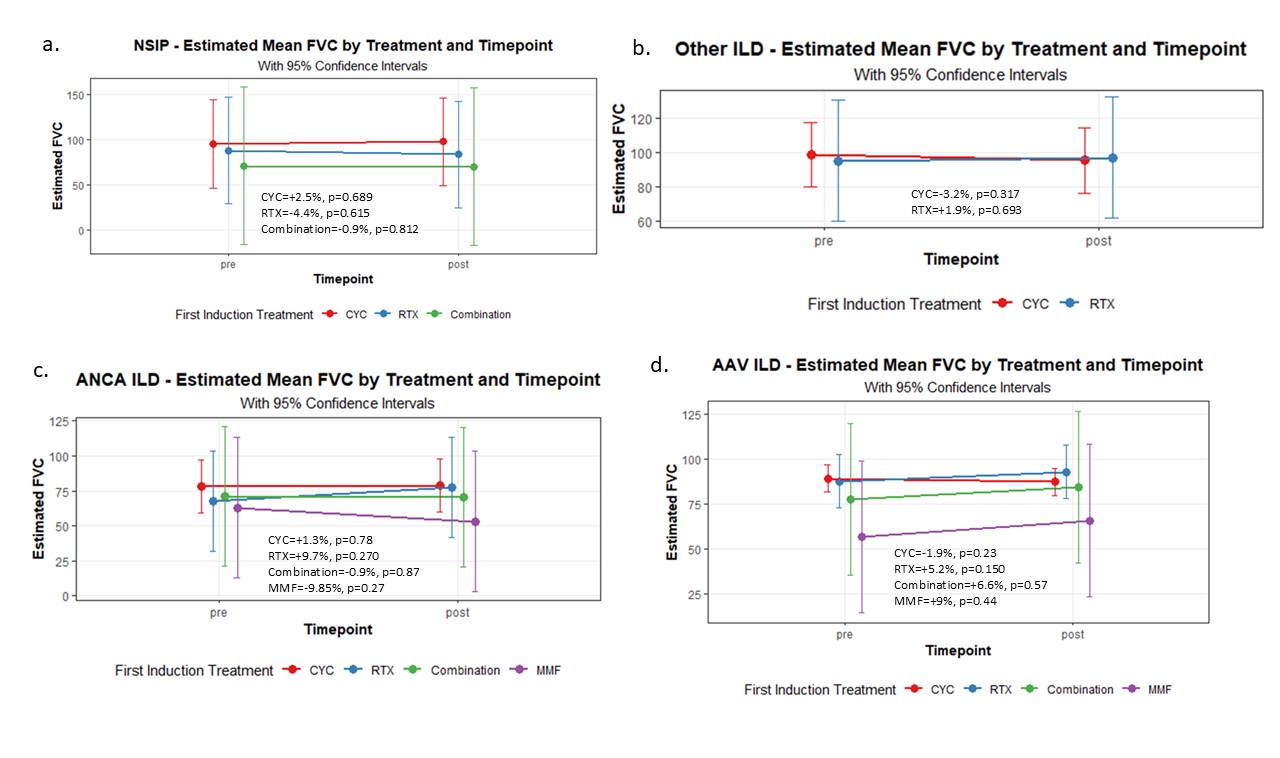


Dots represent the marginal mean values, as estimated by a mixed effects model adjusted for age, gender treatment naivety, with vertical lines representing 95% confidence intervals

CYC; cyclophosphamide, RTX; rituximab, combination; CYC+RTX, MMF; mycophenolate mofetil

**Supplemental Table S8.** Logistic Regression for Force Vital Capacity (FVC) major decline (>10%) after the first year

| Variables | FVC% decline >10% | | | |
| --- | --- | --- | --- | --- |
|  | Events  n | OR | 95% CI | P value |
| Age at AAV diagnosis | 17 | 0.99 | 0.95 to 1.05 | 0.79 |
| Gender  Male (ref.)  Female | 17 | -  0.56 | -  0.18 to 1.65 | 0.3 |
| BMI | 16 | 1.07 | 0.96 to 1.18 | 0.22 |
| ANCA type  MPO-ANCA (ref.)  PR3-ANCA | 17 | -  0 | - | **0.012** |
| ANCA levels at diagnosis | 17 | 1 | 1.00.1 | 0.14 |
| Phenotype  MPA (ref.)  GPA  EGPA  No AAV | 17 | -  0  0  0.34 | -  0.07 to 1.19 | **0.036** |
| Radiological Pattern  UIP (Ref.)  NSIP  others | 11 | -  1.31  0.7 | -  0.17 to 6.96  0.10 to 3.39 | 0.85 |
| Fibrosis Severity Grade  0 (Ref)  1  2  3  4 | 11 | -  0.36  1.05  1  3.33 | -  0.01 to 10.2  0.12 to 23.1  0.10 to 22.8  0.22 to 93.0 | 0.58 |
| Radiological Progression (no Ref) | 11 | 0.72 | 0.19 to 2.77 | 0.62 |
| FVC% predicted baseline | 17 | 1 | 0.97 to 1.02 | 0.87 |
| TLCO% predicted baseline | 13 | 0.98 | 0.95 to 1.01 | 0.25 |
| FEV1 baseline | 10 | 1.03 | 0.97 to 1.10 | 0.43 |
| TLC baseline | 4 | 0.95 | 0.87 to 1.01 | 0.12 |
| DAH (no Ref.) | 14 | 3.8 | 0.85 to 15.9 | 0.079 |
| Organ Involvement  Kidney involvement (no Ref.)  Eyes (no Ref)  ENT (no Ref)  Skin (no Ref)  Heart (no Ref)  GI (no Ref)  PNS (no Ref) | 17 | 3.08  0.31  0.52  0.55  27,2  0  0.84 | 1.03 to 10.1  0.02 to 1.79  0.08 to 2.21  0.03 to 3.57  0.00 to Inf  0.17 to 3.11 | **0.045**  0.40  0.64  0.57  0.081  0.48  0.8 |
| eGFR baseline ml/min/1.73m2 | 11 | 0.94 | 0.95 to 1.02 | 0.35 |
| Induction Treatment  CYC (ref)  RTX  Combination  MMF  No treatment | 17 | -  0.56  2.24  1.12  0 | -  0.08 to 2.49  0.27 to 15.3  0.15 to 5.72 | 0.26 |
| Anti-fibrotic Treatment (no Ref) | 17 | 69,9 | 0.00 to Inf | **0.01** |

OR=odds ratio, CI=Confidence Interval, n; number, ref; reference, AAV; ANCA-associated Vasculitis, BMI; Body mass index, MPO; Myeloperoxidase, PR3; proteinase 3; MPA; Microscopic polyangiitis, GPA; Granulomatosis with polyangiitis, EGPA; Eosinophilic granulomatosis with polyangiitis, ENT; Ear, Nose, Throat, GI; Gastrointestinal, PNS; Peripheral Nervous System, FVC; Forced Vital Capacity, TLCOc; carbon monoxide transfer factor corrected for hemoglobin, eGFR; estimated glomerular filtration rate, FEV1; forced expiratory volume in 1 second, TLC; total lung capacity, DAH; diffuse alveolar haemorrhage, UIP, usual interstitial lung disease, NSIP; Non-Significant Interstitial Pneumonia pattern, MMF; mycophenolate mofetil

**Supplemental Table S9**. Logistic Regression for radiological progression

| Variables | Radiological Progression  Univariate Logistic Analysis | | | |
| --- | --- | --- | --- | --- |
|  | Event  n | OR | 95% CI | P value |
| Age at AAV diagnosis | 49 | 0.94 | 0.89 to 0.98 | **0.015** |
| Gender  Male (ref.)  Female | 55 | -  1.88 | 0.84 to 4.34 | 0.13 |
| BMI | 43 | 1.02 | 0.95 to 1.11 | 0.57 |
| ANCA type  MPO-ANCA (ref.)  PR3-ANCA | 55 | -  0.63 | 0.21 to 1.85 | 0.40 |
| ANCA levels at diagnosis | 52 | 1.00 | 1.00 to 1.01 | 0.66 |
| Diagnosis  ANCA-ILD (ref)  AAV-ILD | 55 | -  1.20 | 0.49 to 2.94 | 0.69 |
| Radiological Pattern  UIP (Ref.)  NSIP  others | 50 | -  0.50  0.53 | 0.16 to 1.50  0.20 to 1.35 | 0.22  0.18 |
| Fibrosis Severity Grade  0 (Ref)  1  2  3  4 | 55 | -  0.07  0.23  0.29  0.04 | 0.00 to 0.49  0.01 to 1.51  0.01 to 2.03  0.00 to 0.35 | **0.021**  0.19  0.28  **0.012** |
| FVC% predicted baseline | 49 | 1.00 | 0.98 to 1.02 | 0.83 |
| TLCO% predicted baseline | 40 | 0.99 | 0.97 to 1.02 | 0.46 |
| FEV1 baseline | 33 | 1.01 | 0.96 to 1.06 | 0.68 |
| TLC baseline | 17 | 1.03 | 0.98 to 1.08 | 0.25 |
| Organ Involvement  Kidney involvement (no Ref.)  Eyes (no Ref)  ENT (no Ref)  Skin (no Ref)  GI (no Ref)  PNS | 50 | 1.18  1.27  1.88  1.56  0.92  1.04 | 0.53 to 2.65  0.41 to 4.16  0.65 to 5.92  0.48 to 5.54  0.04 to 23.7  0.36 to 3.04 | 0.68  0.68  0.26  0.47  0.95  0.94 |
| eGFR baseline ml/min/1.73m2 | 23 | 1.0 | 0.97 to 1.02 | 0.70 |
| Induction Treatment  CYC (ref)  RTX  Combination  MMF  No treatment | 54 | -  1.62  0.25  0.98  0.37 | 0.52 to 5.67  0.03 to 1.17  0.20 to 5.35  0.07 to 1.53 | 0.42  0.10  0.98  0.18 |
| Anti-fibrotic Treatment (no Ref) | 54 | 0.26 | 0.01 to 2.15 | 0.26 |

OR=odds ratio, CI=Confidence Interval, n; number, ref; reference, AAV; ANCA-associated Vasculitis, BMI; Body mass index, MPO; Myeloperoxidase, PR3; proteinase 3; ENT; Ear, Nose, Throat, GI; Gastrointestinal, PNS; Peripheral Nervous System, FVC; Forced Vital Capacity, TLCOc; carbon monoxide transfer factor corrected for hemoglobin, eGFR; estimated glomerular filtration rate, FEV1; forced expiratory volume in 1 second, TLC; total lung capacity, UIP, usual interstitial lung disease, NSIP; Non-Significant Interstitial Pneumonia pattern, MMF; mycophenolate mofetil

**Supplemental Table S10.** Univariate Cox regression of fibrosis severity grade with overall and respiratory survival

|  | **Survival** | | | **Respiratory Survival** | | |
| --- | --- | --- | --- | --- | --- | --- |
| Severity Fibrosis Grade | HR | 95% CI | p-value | HR | 95% CI | p-value |
| 1 | Reference |  |  | Reference |  |  |
| 2 | 1.88 | 0.84, 4.20 | 0.13 | 7.42 | 0.85, 64.7 | 0.070 |
| 3 | 2.41 | 1.05, 5.54 | **0.038** | 10.2 | 1.22, 86.2 | **0.032** |
| 4 | 5.84 | 2.17, 15.7 | **<0.001** | 75.1 | 7.13, 791 | **<0.001** |
| HR=Hazard Ratio, CI=Confidence Interval | | | | | | |

**Supplemental Table S11.** Univariate Cox regression of overall and respiratory survival

| Variables | Survival | | | |  | Respiratory survival | | |
| --- | --- | --- | --- | --- | --- | --- | --- | --- |
|  | Events  N | HR | 95% CI | P value | Events  N | HR | 95% CI | P value |
| Age at AAV diagnosis | 143 | 1.08 | 1.04 to 1.11 | **<0.001** | 89 | 1.00 | 0.96 to 1.05 | 0.92 |
| Gender (male ref.)  Female | 145 | 0.95 | 0.59 to 1.52 | 0.83 | 89 | 2.30 | 0.86 to 6.15 | 0.10 |
| BMI | 103 | 0.96 | 0.91 to 1.01 | 0.11 | 79 | 0.96 | 0.88 to 1.05 | 0.38 |
| Respiratory failure (no ref.) | 144 | 1.89 | 1.14 to 3.14 | **0.014** |  |  |  |  |
| ANCA type (MPO-ANCA ref.)  PR3-ANCA | 145 | 0.76 | 0.4 to 1.46 | 0.410 | 89 | 0.24 | 0.03 to 1.85 | 0.17 |
| ANCA levels at diagnosis | 139 | 1.00 | 1.00 to 1.00 | **0.003** | 87 | 1.00 | 0.99 to 1.01 | 0.43 |
| Phenotype (MPA ref.)  GPA  EGPA  No AAV | 145 | 0.69  1.61 | 0.36 to 1.34  0.82 to 3.17 | 0.280  0.170 | 89 | 0.41  3.83 | 0.05 to 5.27  1.33 to 3.25 | 0.34  **0.013** |
| Radiological Pattern  UIP (Ref.)  NSIP  others | 93 | -  0.35  0.43 | 0.11 to 1.14  0.21 to 0.89 | 0.081  0.023 | 69 | -  0.91  0.26 | 0.20 to 4.21  0.06 to 1.17 | 0.90  0.079 |
| Fibrosis Severity Grade  0 (Ref)  1  2  3  4 | 98 | -  69.5  94.6  121.5  293.9 | (0.00 to Inf)  (0.00 to Inf)  (0.00 to Inf)  (0.00 to Inf) | >0.99  >0.99  >0.99  >0.99 | 73 | -  37.64  190.5  262.8  1.932.8 | 0.00 to Inf  0.00 to Inf  0.00 to Inf  0.00 to Inf | >0.99  >0.99  >0.99  >0.99 |
| Radiological Progression (no Ref) | 94 | 0.54 | 0.28 to 1.02 | 0.059 | 73 | 1.11 | 0.37 to 3.33 | 0.85 |
| FVC% predicted baseline | 101 | 0.97 | 0.96 to 0.99 | **<0.001** | 73 | 0.95 | 0.92 to 0.97 | **<0.001** |
| TLCO% predicted baseline | 84 | 0.98 | 0.97 to 1.00 | **0.02** | 57 | 0.97 | 0.93 to 1.00 | 0.052 |
| FEV1 baseline | 66 | 1.00 | 0.96 to 1.04 | 0.940 | 66 | 1.05 | 0.99 to 1.11 | 0.13 |
| TLC baseline | 37 | 0.94 | 0.91 to 0.98 | **0.002** | 37 | 0.91 | 0.85 to 0.96 | **0.002** |
| DAH (no Ref.) | 128 | 0.50 | 0.24 to 1.07 | 0.074 | 79 | 0.27 | 0.04 to 2.03 | 0.20 |
| Organ Involvement  Kidney involvement (no Ref.)  Eyes (no Ref)  ENT (no Ref)  Skin (no Ref)  Heart (no Ref)  GI (no Ref)  PNS (no Ref) | 139 | 0.92  0.27  0.48  0.50  2.65  0.46  0.94 | 0.55 to 1.53  0.08 to 0.86  0.24 to 0.98  0.23 to 1.11  0.64 to 10.9  0.06 to 3.39  0.47 to 1.86 | 0.740  **0.027**  **0.045**  0.087  0.18  0.45  0.86 | 88 | 0.44  0.28  0.62  0.65  0.00  0.00  0.79 | 0.16 to 1.16  0.04 to 2.09  0.17 to 2.21  0.15 to 2.85  0.00 to Inf  0.00 to Inf  0.22 to 2.79 | 0.10  0.21  0.46  0.57  >0.99  >0.99  0.71 |
| eGFR baseline | 81 | 0.99 | 0.97 to 1.00 | 0.160 | 38 | 1.03 | 0.98 to 1.08 | 0.22 |
| Induction Treatment  CYC (ref)  RTX  Combination  MMF  No treatment | 141 | -  1.86  1.83  0.54  0.00 | 1.02 to 3.37  0.88 to 3.82  0.16 to 1.77  0.00 to Inf | **0.041**  0.11  0.31  >0.99 | 89 | -  2.98  1.55  2.50  0.00 | 1.05 to 8.43  0.33 to 7.21  0.30 to 20.9  0.00 to Inf | **0.040**  0.57  0.40  >0.99 |
| Anti-fibrotic Treatment (no Ref) | 141 | 2.08 | 0.50 to 8.61 | 0.31 |  | 6.19 | 1.39 to 27.5 | **0.017** |

HR=Hazard Ratio, CI=Confidence Interval, n; number, ref; reference, AAV; ANCA-associated Vasculitis, BMI; Body mass index, MPO; Myeloperoxidase, PR3; proteinase 3; MPA; Microscopic polyangiitis, GPA; Granulomatosis with polyangiitis, EGPA; Eosinophilic granulomatosis with polyangiitis, ENT; Ear, Nose, Throat, GI; Gastrointestinal, PNS; Peripheral Nervous System, FVC; Forced Vital Capacity, TLCOc; carbon monoxide transfer factor corrected for hemoglobin, eGFR; estimated glomerular filtration rate, FEV1; forced expiratory volume in 1 second, TLC; total lung capacity, DAH; diffuse alveolar haemorrhage, UIP, usual interstitial lung disease, NSIP; Non-Significant Interstitial Pneumonia pattern, MMF; mycophenolate mofetil, GCs; Glucocorticoids, AZA; azathioprine, MTX; methotrexate

**Supplemental Table S12.** Causes of death

| **Causes of death** | **Total cohort**  ***n*=78** |
| --- | --- |
| Lung infection/ ILD exacerbation % (n) | 44 (34) |
| Other infection, % (n)  Sepsis, % (n)  VZV, % (n)  Diverticulitis, % (n) | 5 (4)  2 (2)  1 (1)  1 (1) |
| MACE, % (n) | 9 (7) |
| Malignancy, % (n)  Lung, % (n)  Pancreas, % (n) | 4 (3)  2 (2)  1 (1) |
| Not known, % (n) | 29 (23) |

n; number, VZV; Varicella-Zoster Virus, MACE; Major adverse cardiovascular events
